# Searching for Signatures of Cold Climate Adaptation in *TRPM8* Gene in Populations of East Asian Ancestry

**DOI:** 10.3389/fgene.2019.00759

**Published:** 2019-08-23

**Authors:** Alexander V. Igoshin, Konstantin V. Gunbin, Nikolay S. Yudin, Mikhail I. Voevoda

**Affiliations:** ^1^Sector of the Genetics of Industrial Microorganisms, The Federal Research Center Institute of Cytology and Genetics, The Siberian Branch, The Russian Academy of Sciences, Novosibirsk, Russia; ^2^Center of Brain Neurobiology and Neurogenetics, The Federal Research Center Institute of Cytology and Genetics, The Siberian Branch, The Russian Academy of Sciences, Novosibirsk, Russia; ^3^V. Zelman Institute for Medicine and Psychology Novosibirsk State University, Novosibirsk, Russia; ^4^Center for Mitochondrial Functional Genomics, Institute of Living Systems, Immanuel Kant Baltic Federal University, Kaliningrad, Russia; ^5^Laboratory of Livestock Molecular Genetics and Breeding, The Federal Research Center Institute of Cytology and Genetics, The Siberian Branch, The Russian Academy of Sciences, Novosibirsk, Russia; ^6^Laboratory of Human Molecular Genetics, The Federal Research Center Institute of Cytology and Genetics, The Siberian Branch, The Russian Academy of Sciences, Novosibirsk, Russia

**Keywords:** TRPM8, environmental correlation analysis, SNP, cold adaptation, East Asian ancestry

## Abstract

Dispersal of *Homo sapiens* across the globe during the last 200,000 years was accompanied by adaptation to local climatic conditions, with severe winter temperatures being probably one of the most significant selective forces. The *TRPM8* gene codes for a cold-sensing ion channel, and adaptation to low temperatures is the major determinant of its molecular evolution. Here, our aim was to search for signatures of cold climate adaptation in *TRPM8* gene using a combined data set of 19 populations of East Asian ancestry from the 1000 Genomes Project and Human Genome Diversity Project. As a result, out of a total of 60 markers under study, none showed significant association with the average winter temperatures at the locations of the studied populations considering the multiple testing thresholds. This might suggest that the principal mode of *TRPM8* evolution may be different from widespread models, where adaptive alleles are additive, dominant or recessive, at least in populations with the predominant East Asian component. For example, evolution by means of selectively preferable epistatic interactions among amino acids may have taken place. Despite the lack of strong signals of association, however, a very promising single nucleotide polymorphism (SNP) was found. The SNP rs7577262 is considered the best candidate based on its allelic correlations with winter temperatures, signatures of selective sweep and physiological evidences. The second top SNP, rs17862920, may participate in adaptation as well. Additionally, to assist in interpreting the nominal associations, the other markers reached, we performed SNP prioritization based on functional evidences found in literature and on evolutionary conservativeness.

## Introduction

Recent paleoanthropological evidences show the presence of anatomically modern humans in Africa as early as 300 kya (Hublin et al., 2017), with the earliest known “Out of Africa” migration event dating back to 200 kya (Hershkovitz et al., 2018). Dispersal of *Homo sapiens* across the globe during the last 200,000 years was accompanied by adaptation to local environments. Spatial variations in selective pressures have ultimately led to observable geographic distribution of many physiological and anatomical traits in present-day humans. For example, the low level of UV radiation at higher latitudes is now considered to be the major cause of evolution of depigmented skin (Jablonski and Chaplin, 2010).

Since the mid-2000s, there has been significant progress in genotyping technologies, followed by publicly available databases of human genetic variation. This circumstance helped the population geneticists to discover signatures of human local adaptation from genome-wide genotyping data. Human microevolution driven by the action of low temperatures has long been attracting attention of the scientific community. Now, a number of studies have been dedicated to this issue both at the genome level (e.g., Hancock et al., 2011a; Cardona et al., 2014; Valverde et al., 2015) and at the level of selected regions or genes (Hancock et al., 2008; Ohashi et al., 2011; Hancock et al., 2011b; Sazzini et al., 2014; Quagliarello et al., 2017).

Probably the best-known gene in terms of its possible role in adaptation to cold climate is *TRPM8* located on human chromosome 2. This gene codes for ion channel functioning as a thermal sensor, detecting temperatures in the range from 15 to 30°C (Fernández et al., 2011). There are evidences supporting its physiological role in thermoregulation, and in fact, TRPM8 is the only well-established cold receptor in mammals (Bautista et al., 2007; Colburn et al., 2007; Dhaka et al., 2007). Besides these, there are data on associations of its single nucleotide polymorphisms (SNPs) with sensitivity to cold (Kozyreva et al., 2011), the respiratory system response to cooling (Kozyreva et al., 2014), blood lipids, and anthropometric parameters in humans (Potapova et al., 2014). The *TRPM8* gene was suggested to underlie genetic adaptation to cold in ground squirrel and hamster (Matos-Cruz et al., 2017), sheep (Fariello et al., 2014; Liu et al., 2016), and humans (Cardona et al., 2014; Key et al., 2018). According to modern views, adaptation to low temperatures is the major determinant of *TRPM8* molecular evolution (Myers et al., 2009; Majhi et al., 2015).

To our knowledge, a study by Key and colleagues (2018) is the only one to use environmental data to search for signatures of cold climate adaptation in the *TRPM8* gene. The authors used latitudes and annual average temperatures at the locations of the populations of the Old World as predictors for SNP allele frequency distributions. They found evidences that SNP rs10166942 had undergone climate-mediated selection, which raised its derived allele frequency from south to north.

In our opinion, focusing on closely related populations is preferable to using large population sets for the following reasons. First, the ability to survive in a severely cold climate is supposed to be highly polygenic, as many biological processes like vasoconstriction, nonshivering thermogenesis, regulation of adipocyte differentiation, and thermoception are expected to be involved. It is known that the adaptation to cold can be associated with quite different genetic bases (Yudin et al., 2017). Because different branches of *Homo sapiens* are likely to have had distinct genetic background before and during the process of climate-driven selection, it is possible that in phylogenetically distant groups, adaptation may have recruited different genes. Second, even in the case when selection is acting on the same gene, variants involved in adaptation may differ in different branches. Our supposition is supported by the example of variants associated with lactase persistence. Thus, within European populations, the activity of the lactase enzyme in adulthood is connected with the C/T-13910 variant in the enhancer region of the *LCT* gene, whereas in sub-Saharan Africa, this trait is mainly correlated with the presence of the G/C-14010 mutation (Tishkoff et al., 2007). Therefore, it would be sensible to search for microevolution in clusters of related populations.

For the above reasons, the aim of this study was to search for signatures of adaptation to low temperatures in the *TRPM8* gene under various null hypotheses of population structure and dynamics using a combined data set of 19 populations of the East Asian ancestry from Human Genome Diversity Project (HGDP) and 1000 Genomes (1000G) Project with the assistance of environmental correlation analysis techniques. Locations of chosen populations are characterized by a large range of average winter temperatures (−37–+27°C), implying substantial differences in selection pressures.

## Materials and Methods

### Genotypic and Environmental Data

In this study, we used genotypic data on 656 individuals from 19 HGDP (Cann et al., 2002) and 1000G Project (1000 Genomes Project Consortium et al., 2010) populations (**Supplementary Table 1**) having predominantly the East Asian genetic component. Data on SNPs belonging to *TRPM8* gene were obtained from NCBI dbSNP (https://www.ncbi.nlm.nih.gov/snp/), resulting in 60 polymorphic markers (minor allele frequency >0.01) being at the intersection of HGDP and 1000G sets. The missing genotypes in HGDP data were imputed using fastPHASE v.1.4.8 software (Scheet and Stephens, 2006) with default parameters. Genotypic information from HGDP and VCF formats was combined by using a self-made Python 3 script, so that inconsistency of DNA strands between databases (if that was the case) was resolved using 1000G VCF as a reference. Besides *TRPM8* SNPs, we used 5 Mb regions upstream and downstream this gene (1,309 markers at r^2^ < 0.7) to infer the phylogenetic tree used in PGLS, to estimate the covariance matrix used in Bayenv2-BLM and Bayenv2-SRC, to correct for background levels of the population structure in LFMM and to make population inferences in BayScEnv more precise. Also, this set of SNPs was used to construct a null distribution for empirical p-value calculation. Information on latitudes and longitudes for 1000G populations was taken from Key et al. (2018). Latitudes and longitudes for HGDP populations were taken from Cann et al. (2002), and average winter temperature values were obtained from ClimateCharts.net database (https://climatecharts.net) using the corresponding coordinates. We believe the average winter temperature to be a more pertinent predictor for distribution of cold-adaptive alleles than the annual average temperature, as regions with a continental climate may have cold winters and hot summers.

### Construction of a Phylogenetic Tree and Statistical Analysis

As our sample consisted of phylogenetically close populations, we first performed conventional Spearman’s rank correlation test not accounting for the sample structure (Spearman, 1904) at the population (i.e., using allele frequencies) and individual’s (i.e., using allele dosages) levels.

PGLS analysis (R package ‘ape,’ Paradis et al., 2004) was carried out at the individual’s level using the simplest Brownian motion model. We selected this type of analysis because it is an opposite alternative (phyletic evolution) to the conventional Spearman’s rank correlation test. The phylogenetic tree used in this test was reconstructed with IQ-TREE v. 1.5.5 subprogram ModelFinder (Kalyaanamoorthy et al., 2017) based on the best nucleotide substitution model.

Our primary aim was to test the association between the climatic factor and allele frequencies. For this purpose, we chose two independent approaches: the Bayesian linear model from Bayenv2 (further referred to as Bayenv2-BLM) software (Günther and Coop, 2013) and LFMM (Frichot et al., 2013), each building a regression model relating allele frequencies to environmental values. To minimize the problem of false-positive associations between allele frequencies and environment because of the population structure, the above methods take into account allele frequency correlations across populations while performing the analysis by various ways.

In addition, we used Spearman’s rank correlation test from Bayenv2 (further referred to as Bayenv2-SRC) that uses allele frequencies standardized to have no covariance. It is less powerful than Bayenv2-BLM but more robust to outliers and can detect monotonic relationships.

BayScEnv test (de Villemereuil and Gaggiotti, 2015) was used as an alternative to Bayenv2-BLM and LFMM. This method assumes that all populations are independent and exchange genes through the limited migrant pool; it includes a locus-specific effect unrelated to the environmental variable, taking into consideration locus-specific deviations from a neutral model. BayScEnv software was also used to calculate F_st_ distances for each locus averaged over populations (**Supplementary Table 2**).

In addition to the correlation techniques, we tried the XP-CLR test (Chen et al., 2010) as a complementary approach. This test is designed for detecting selective sweeps on the basis of joint modeling of the multilocus allele frequency differentiation between two populations. The method does not require information on the ancestral/derived status at each SNP (Chen et al., 2010; Vatsiou et al., 2016).

For more details on phylogenetic tree construction and statistical analysis, please see the Supplementary Material.

### SNP Prioritization

We prioritized SNPs based on three types of evidences found in literature, “association with trait relevant to survival in a cold climate,” “evidences for cold-mediated selection,” and “association with any other phenotype or risk.” These categories were given weights 3, 2, and 1, respectively, and a score for each SNP was summarized (**Supplementary Table 3**). The key assumption behind this prioritization is that because of the pleiotropic nature of *TRPM8* gene, allelic substitutions having any functional manifestation may potentially have more chances of affecting survival in cold climate conditions than those not having any known effects.

Additionally, we obtained the PhyloP100way vertebrate conservation score for each SNP (**Supplementary Table 4**) from UCSC Genome Browser (Casper et al., 2018). Currently, it is commonly thought that the genetic drift plays a minor role in the evolution of conservative sites, and relatively rare allele replacements occurring therein are mostly driven by positive selection (Andolfatto, 2005; Cai et al., 2009; Halligan et al., 2011; Bazykin and Kondrashov, 2012). Therefore, significant allelic correlation with environmental gradient supported by a high conservation score promises to be the true sign of local adaptation.

## Results

Contrary to what we had expected, only three SNPs out of a total of 60 markers under study showed nominally significant association with the average winter temperatures at the locations of the studied populations by any two types of analysis (the results of tests carried out using default/recommended parameters are shown in Table 1; for full results, see **Supplementary Tables 5 and 6**). When considering the multiple testing threshold, however, none of them is significant (adjusted p values not shown).

**Table 1:** Genic and upstream *TRPM8* variants showing nominally significant (in bold) association in at least two correlation tests with default/recommended parameters (K = 2 for LFMM and pi = 0.1/p = 0.5 for BayScEnv).

Methods and scores	rs7577262	rs17862920	rs6723922	rs11682848
SNP evidence score	NA*	**5**	**1**	**1**
PhyloP100way conservation score	**−1.7724**	**−0.1171**	**−2.2644**	**−5.512**
**Bayenv2-BLM empirical p-value**	**0.0374**	0.06	0.064	**0.0069**
**PGLS p-value**	**0.0331**	**0.04**	0.101	0.121
**Bayenv2-SRC empirical p-value**	**0.0252**	**0.0328**	0.075	**0.0145**
**SRC individual-based empirical p-value**	**0.0267**	0.054	0.096	**0.0481**
**SRC population-based empirical p-value**	**0.042**	0.149	**0.0428**	0.102
LFMM p-value	**0.074**	0.127	**0.005**	**0.0013**
**BayScEnv posterior error probability/q-value**	0.97/0.829	0.984/0.903	0.993/0.957	0.795/0.525
**BayScEnv empirical p-value**	**0.042**	0.084	0.208	**0.0115**

*The SNP evidence score was not calculated for rs7577262 because of a high level of confidence in its adaptive role.

SNP rs11682848 was previously reported as associated with the prognosis of colorectal cancer (Walther, 2010). Interestingly, such connection of climate-associated loci with cancer has already been noticed by other researchers (Hancock et al., 2011a). Furthermore, it has been recently shown by combination of 247 genome-wide association studies that cold selected genes are enriched with cancer-associated genes (Voskarides, 2018). Ironically, SNP rs11682848 has the lowest conservation score among 60 markers under study. This means that either rs11682848 is being a false-positive finding or being linked to some functional variant.

SNP rs17862920 has evidences of associations with migraine susceptibility (Freilinger et al., 2012; Meng et al., 2018). The rs17862920-С allele predisposing to migraine is more prevalent in northern latitudes. Also, rs17862920 has been shown to be associated with sensing cold pain in Finnish and Norwegian individuals, with C allele carriers being more susceptible (Kaunisto et al., 2013). Migraine has been reported to be related to increased pain perception of nonnoxious cold temperatures (Burstein et al., 2000). Unlike rs11682848, PhyloP100way conservation score for rs17862920 is more promising and has a rank of 20/60 while still being negative. It is possible that allele substitution in rs17862920 has a functional effect. Thus, rs17862920 was predicted to regulate *TRPM8* transcription by TFsearch and GoldenPath in F-SNP bioinformatics tool (Ghosh et al., 2013).

As for rs6723922, this SNP is a genetic risk factor for severe cutaneous adverse drug reactions (Park et al., 2018). One could hypothesize that there is a certain mechanism underlying both the altered cold sensation and the increased cutaneous susceptibility to chemicals. It could be no surprise given that the TRPM8 channel is activated by a variety of chemical ligands (Beccari et al., 2017). Like rs11682848, SNP rs6723922 has a low conservation score (the rank of 55/60), implying conclusions for this marker similar to those for rs11682848.

The results of the XP-CLR test are more encouraging (**Supplementary Figure S1**). It appears that there is a pronounced trend for several pairs of populations to show the signature of a selective sweep 10 ± 6 Kb upstream from the *TRPM8* gene. The direction of selection in this region is seen when reversing tested and reference populations in pairs (e.g., compare “JPT vs. KHV” and “KHV vs. JPT”). The strongest XP-CLR peaks within this putative sweep are mainly located near rs10929317 and rs7577262 SNP loci. The former was removed from the analysis because of high LD with rs17862920: r^2^ = 0.966/*D’* = 0.995 in East Asian populations (LDlink tool; Machiela and Chanock, 2015) and is therefore expected to be as significant as rs17862920. The latter was used in the control set of 1,309 markers. Surprisingly, this SNP demonstrates significant association with our climatic variable in almost all of the correlation tests (Table 1). Also, rs7577262 has been reported to be associated with susceptibility to migraine (Anttila et al., 2013) and blood pressure response to the cold pressor test (He et al., 2013).

## Discussion

A variety of facts have led us to think of *TRPM8* gene as being under intense positive selection. We expected that the large amount of SNPs in *TRPM8* would demonstrate strong signals because of being under selection immediately or being linked to some causal variants. However, this is not the case. Furthermore, SNPs detected do not pass the corrected threshold, considering multiple testing. Among possible explanations are the following hypotheses:

1. The predominant mode of *TRPM8* evolution may be different from the widespread models exploited by our tests, where adaptation is assumed to be mediated by selecting alleles with additive or at least recessive/dominant trait coding. However, it is suggested that epistatic interactions may play a role in the evolution of thermoTRP channels (Saito and Tominaga, 2017). Therefore, conformational epistasis-based evolution, where some epistatic interactions among amino acids are preferred, might have resulted in the inability of approaches we used to detect strong signatures of selection in the *TRPM8* gene.
2. The level of allele frequency variation (see **Supplementary Table 7** for minor allele frequency distributions) in the populations studied is not sufficient to robustly discriminate the loci under selection. Thus, the averaged F_st_ distances for populations used by Key et al. (2018) are much higher than those for our data set (**Supplementary Table 2**).
3. Only 5 out of 19 locations of populations from our data set have the average winter temperature below −10°C. It is possible that the underrepresentation of northern populations in this study might lead to insufficient signal strength. Further accumulation of open access data on genetic variation in the north would help in detecting loci under selection.

**Figure 1:**
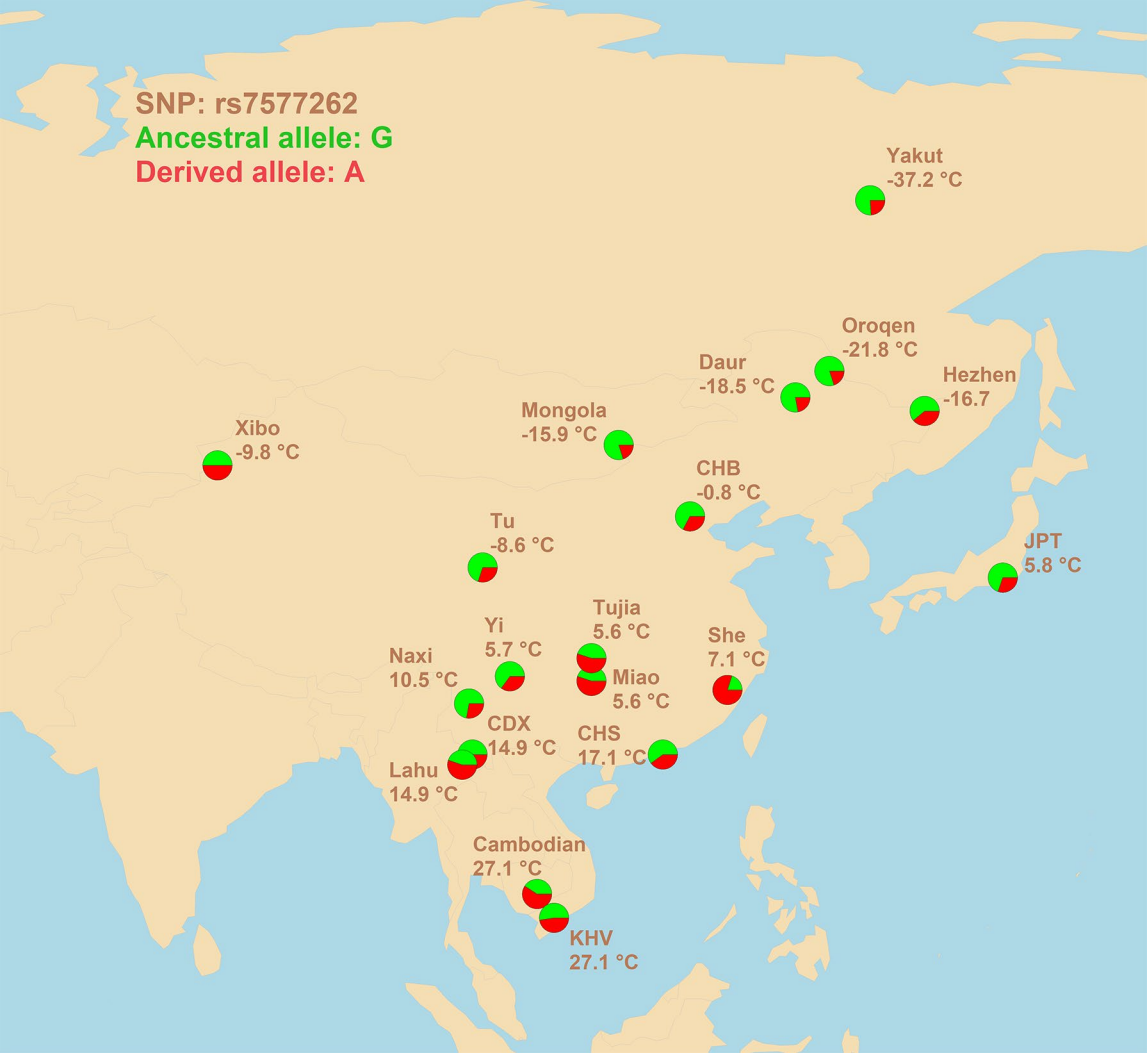
Geographic distribution of allele frequencies for rs7577262 polymorphism in populations of East Asian ancestry. Average winter temperatures at the locations of the populations studied are shown. 1000 Genomes population: CHB, Han Chinese in Beijing, China; JPT, Japanese in Tokyo, Japan; CDX, Chinese Dai in Xishuangbanna, China; CHS, Han Chinese South, China; KHV, Kinh in Ho Chi Minh City, Vietnam.

Despite the lack of strong signals of association, however, a very promising candidate SNP was found. SNP rs7577262 is 7.1 kb upstream of the transcription start site for *TRPM8* mRNA, implying its possible involvement in transcriptional regulation. In addition to correlations and signatures of sweep, physiological data contribute equally to the evidences in favor of selection acting on rs7577262. The rs7577262-G allele is associated with a higher blood pressure response to the cold pressor test (He et al., 2013). It is known that the blood pressure response to the cold pressor test primarily stems from alpha-adrenergically mediated peripheral vasoconstriction (Leppäluoto and Hassi, 1991; Larra et al., 2015), which is, in turn, one of the basic mechanisms of cold adaptation (Daanen and Lichtenbelt, 2016). Given that this allele is more prevalent in northern latitudes (**Figure 1**), its adaptive role may be assumed.

Another SNP, rs17862920, is linked with rs7577262 (r^2^ = 0.59 in East Asians). Probably, this is the reason for the correlation the former demonstrates. Both SNPs are risk loci for migraine. At the same time, it has been mentioned above that rs17862920 is associated with sensing cold pain in Finnish and Norwegian individuals, with C allele carriers (more prevalent in northern latitudes) being more susceptible. It can be assumed that both loci are independently involved in adaptation to low temperatures. In that case, however, the adaptive role of rs17862920-C allele is hard to explain. The possible mechanism of differential survival might be avoidance of potentially lethal hypothermia by those harboring С allele.

As for rs6723922 and rs11682848 loci, none of them shows any sign of selective sweep in the XPCLR test. Probably, those are false positives, or at least, linked to an unobserved variant under selection. It is also worth noting that SNP evidence scores for these loci are quite low.

In addition to the search for signatures of selection, we would like to note some details on the BayScEnv test not published anywhere (as far as we know).

Changing model parameters drastically affects the output in BayScEnv (see **Supplementary Table 6**). For example, significant results (q value <0.05) were obtained when using model parameters pi = 0.5/p = 0.1 (SNP rs11682848 being significant) or pi = 0.9/p = 0.1 (18/60 SNPs being significant). At the same time, empirical p values are more stable. Thus, we suggest using them in hypothesis-driven studies (with default model parameters) of local adaptation and choosing the significance threshold based on expert’s opinions rather than relying on FDR outputs.

Counterintuitively, a reduction in the number of tests in BayScEnv does not lead to a greater number of statistically significant FDR outputs (the posterior error probability and the q value). Furthermore, in our case, given parameters pi = 0.9/ p = 0.1, 18 out of a total of 60 SNPs reach significance level when analyzing 1,369 markers, whereas none is significant when using 60 SNPs. This discrepancy might be explained by less precise constructing a null model of population structure.

## Conclusions

Several lines of evidence point to possible involvement of rs7577262 in cold adaptation. This SNP is considered the best candidate based on its allelic correlations with winter temperatures, signatures of selective sweep and physiological evidences. The second top SNP, rs17862920, may participate in adaptation as well. As for rs6723922 and rs11682848 loci, these appear to be false positives or at least linked to some unobserved selected variant.

## Author Contributions

NY and MV conceived the project. KG supervised the project. AI and KG processed and analyzed the data. AI, KG, and NY drafted the manuscript.

## Funding

This study was supported by budget from project No. 0324-2019-0041 of the Federal Research Center «Institute of Cytology and Genetics» SB RAS (ICG SB RAS).

## Conflict of Interest Statement

The authors declare that the research was conducted in the absence of any commercial or financial relationships that could be construed as a potential conflict of interest.
