# Supplementary material for: Searching for Signatures of Cold Climate Adaptation in TRPM8 Gene in Populations of East Asian Ancestry
Source: Front Genet. 2019 Aug 23;10:759. doi: 10.3389/fgene.2019.00759 (PMC6716346; doi:10.3389/fgene.2019.00759)
Supplement: Supplementary file 1 [file DataSheet_1.zip › SupplementaryMaterials/Supplementary Material.docx]

**Supplementary Material**

**Construction of a phylogenetic tree**

In order to estimate the phylogenetic relationship between human individuals, we converted SNP’s genotype data (PLINK generated files, non-phased data) into a FASTA file containing two nucleotide sequences for each individual. Because we did not have robust and reliable gametic phase information (a fastPhase algorithm was used with the sole intention of imputing missing genotypes), genotypes were treated as non-phased. In the initial non-phased SNP’s genotype data, the first allele is usually a reference allele (except cases of homozygosity by an alternative allele) while the second one is an alternative allele (except cases of homozygosity by a reference allele). Therefore, after conversion of the non-phased genotype data into a FASTA file for each individual we obtained two sequences: the first one corresponds to the homozygous and reference states (we called this sequence “conservative sequence”), while second one reflects heterozygous states enriched with alternative alleles (we called this sequence as “variable sequence”). The conservative sequence reflects the populational ancestry of an individual, while a variable sequence represents the results of genetic admixture between various populations. For phylogenetic reconstruction we used both sequences from each individual and allowed to cluster these sequences independently (we did not use any topological constraints). This allowed us to account for the role of admixture in the phylogeny of every investigated human population explicitly. To fit various models of the evolution of nucleotide sequences, we used IQTree v. 1.5.5 subprogram ModelFinder (Kalyaanamoorthy et al., 2017). The best model of nucleotide substitutions by all statistics (-ln(L), AIC, AICc, BIC) was transversion model TVM+R10, in which base frequencies and transversion rates are variable, while the transition rates are equal. Using this model, we reconstructed the phylogenetic tree (placed at the end of this document).

**Statistical analysis**

**Spearman’s rank correlation**

Because our sample consisted of phylogenetically close populations, we first performed conventional Spearman’s rank correlation test not accounting for sample structure (Spearman, 1904) at the population (i.e. using allele frequencies) and individual (i.e. using allele dosages) levels. P-values for this test were calculated empirically as described below.

**PGLS**

PGLS analysis (R package “ape”, Paradis et al., 2004) was carried out at the individual level using the simplest Brownian motion model. We selected this type of analysis because it is an opposite alternative (phyletic evolution) to the conventional Spearman’s rank correlation test. In order to account for data variability due to possible population admixture, we repeated PGLS analysis several times based on a single randomly chosen sequence (conservative or variable) from each individual. To extract the subtree that corresponds to individual randomly chosen sequences from phylogenetic tree obtained at the stage of “phylogenetic tree construction”, we used newick-utils-1.6 package (Junier and Zdobnov, 2010). The genotype and temperature were treated as dependent and independent variables, respectively. Twenty iterations of PGLS analysis were done, and the harmonic mean of a set of p-values at each SNP was calculated across all runs. In order to correct for inflation of test statistics, we rescaled each statistic value replacing χ^2^ by χ^2^/λ, where λ (λ=2.79) is the inflation factor (the standard genomic control procedure described e.g. in Hinrichs et al., 2009). For λ estimation to be correctly performed, we added p-values of 1309 control SNPs to our test set.

**Bayenv2-BLM**

The Bayenv2-BLM approach attempts to control for the effects of population structure using the Bayesian hierarchical model. A key parameter of the model is the covariance matrix of allele frequencies among populations, which accounts for neutral population structure. Estimation of the covariance matrix was performed with 1369 markers (1309 + 60) by running 100,000 iterations of the Markov Chain Monte Carlo (MCMC) algorithm and averaging across 20 matrix estimates. Then, for each SNP, we ran 100,000 MCMC iterations. Because of relatively low reproducibility of Bayenv2-BLM (Blair et al., 2014), we repeated this procedure 5 times, and the average Bayes factor (BF) across all repetitions was used as a final estimate for each SNP. Because the null distribution of BF is unknown, we could not compute a p-value analytically, therefore, it was calculated empirically by estimating BF for a set of control loci and calculating the probability of observing the particular or more extreme value of BF under this empirical null model. To this end, we used formula recommended by North et al., (2002):

$$p\boldsymbol{=}\frac{(r+1)}{(n+1)}$$

where p is a p-value for SNP of interest, r is the number of SNPs with BF values being greater or equal to BF of SNP tested, n is the overall SNP number.

**Bayenv2-SRC**

We also used Spearman’s rank correlation test from Bayenv2 (further referred to as Bayenv2-SRC) which uses allele frequencies standardized to have no covariance. It is less powerful than Bayenv2-BLM, but more robust to outliers and can detect monotonic relationships. Statistical significance of Spearman's correlation coefficient was calculated empirically as well.

**LFMM**

LFMM analysis was carried out with R package «LEA» (Frichot and Francois, 2015). LFMM tests the correlations between environmental variables and allele frequencies simultaneously inferring the population structure via the so-called latent factors or ancestry coefficients. The number of ancestry coefficients (K=2, see Supplementary Figure S2) was chosen using “snmf” function from “LEA” package. However, we have additionally launched LFMM with the range of various K’s. Inflation factor λ for each run was estimated based on 1369 (1309 + 60) SNPs and p-values were rescaled as described for PGLS analysis. For results to be more stable, the parameter “rep” in LFMM was set to 41.

**BayScEnv**

BayScEnv test (Villemereuil and Gaggiotti, 2015) was used as an alternative to Bayenv2-BLM and LFMM. This method assumes that all populations are independent and exchange genes through the limited migrant pool; it includes a locus specific effect unrelated to the environmental variable, taking into consideration locus specific deviations from a neutral model. BayScEnv test was performed with 1369 (1309 + 60) SNPs using different model parameters (prior probability pi against the neutral model and prior preference p for the locus-specific effect model), in order to find out which mode is more appropriate when testing for local adaptation in predefined candidate genes. The climatic variable used was standardized to have zero mean and unit variance. In addition to FDR outputs produced by BayScEnv, empirical calculation of p-values was performed as well, based on negative q-value logarithms. It is expected that a greater number of loci leads to more accurate estimation of the null model of population structure in BayScEnv. Nonetheless, we additionally ran BayScEnv merely with 60 SNPs from *TRPM8* gene to figure out if this reduction in number of tests significantly influences FDR outputs for SNPs under study.

**XP-CLR**

The Cross-Population Composite Likelihood Ratio (XP-CLR) test (Chen et al., 2010) is designed to test for selection in two separated populations after their divergence. This method models the evolutionary path of an allele under two contrast models (Chen et al., 2010): evolution under neutrality (as Brownian motion of random variable X governed by normal distribution with the given mean and variance) and selective sweep (by applying mathematical transformation to the normal distribution of the random variable X depending on the distance to the selected allele and the strength of selection). After modeling, the method compares the likelihood of the data for each of the two models. XP-CLR is robust to ascertainment bias and does not require information on the ancestral/derived status at each segregating SNPs (Chen et al., 2010; Vatsiou et al., 2016). Despite missing of ancient selective events (Racimo, 2016) XP-CLR is powerful (comparing to HS, nSL, EHHST, xp-EHH, XP-EHHST approaches) in detecting hard and soft selective sweep on a wide range of allele frequencies of selected loci (Vatsiou et al., 2016) and applicable for one of the widest frequency ranges of the beneficial allele (Weigand and Leese, 2018). In our study, XP-CLR scores were computed at regularly spaced grid points (every 2 kb), the sliding window size was 1 cM around the grid points, in order to consider for variation in SNP densities among genomic regions, we restricted the maximal number of SNPs to 200 (if the number of SNPs within a window is more than 200, the exceeded number of SNPs will be randomly removed). For analysis, we chose five 1000 Genomes populations, for their sufficient and comparable sample sizes, and high quality of phased haplotypes. Information on the genetic map distances was taken from <http://bochet.gcc.biostat.washington.edu/beagle/genetic_maps/>. All possible pairs, where one population is CHB/JPT, and another is CDX/CHS/KHV, were investigated in 10 Mb region (*TRPM8* and 5 Mb regions upstream and downstream the gene) comprising 2496 polymorphic loci (with no filtering for high LD). Regions with XPCLR scores that exceed the 95th percentile of the empirical XPCLR distribution were considered as candidates for being under selection.

**References**

Blair, L.M., Granka, J.M., Feldman, M.W. (2014). On the stability of the Bayenv method in assessing human SNP-environment associations. Hum Genomics 8:1. doi: 10.1186/1479-7364-8-1

Chen, H., Patterson, N., Reich, D. (2010). Population differentiation as a test for selective sweeps. Genome Res 20:393-402. doi: 10.1101/gr.100545.109.

de Villemereuil, P., and Gaggiotti, O. E. (2015). A new FST -based method to uncover local adaptation using environmental variables. Methods in Ecology and Evolution 6, 1248–1258. doi: 10.1111/2041-210X.12418

Frichot, E., and François, O. (2015). LEA: an R package for landscape and ecological association studies. Methods in Ecology and Evolution 6, 925–929. doi: 10.1111/2041-210X.12382

Hinrichs, A.L., Larkin, E.K., Suarez, B.K. (2009). Population stratification and patterns of linkage disequilibrium. Genet Epidemiol. 33 Suppl 1, S88-92. doi: 10.1002/gepi.20478.

Junier, T., and Zdobnov, E.M. (2010). The Newick utilities: high-throughput phylogenetic tree processing in the UNIX shell. Bioinformatics. 26, 1669-1670. doi: 10.1093/bioinformatics/btq243

Kalyaanamoorthy, S., Minh, B.Q., Wong, T.K.F., von Haeseler, A., Jermiin, L.S. (2017). ModelFinder: fast model selection for accurate phylogenetic estimates. Nat Methods 14, 587-589. doi: 10.1038/nmeth.4285

North, B.V., Curtis, D., Sham, P.C. (2002). A note on the calculation of empirical P values from Monte Carlo procedures. Am J Hum Genet. 71, 439-441 doi: 10.1086/341527

Paradis, E., Claude, J., Strimmer, K. (2004). APE: Analyses of Phylogenetics and Evolution in R language. Bioinformatics 20, 289-290. doi: 10.1093/bioinformatics/btg412

Racimo, F. (2016). Testing for Ancient Selection Using Cross-population Allele Frequency Differentiation. Genetics. 202:733-50. doi: 10.1534/genetics.115.178095.

Spearman, C. (1904). The proof and measurement of association between two things. Am J Psychol 15, 72-101.

Vatsiou, A.I., Bazin, E., Gaggiotti, O.E. (2016). Detection of selective sweeps in structured populations: a comparison of recent methods. Mol Ecol. 25:89-103. doi: 10.1111/mec.13360.

Weigand, H., Leese, F. (2018). Detecting signatures of positive selection in non-model species using genomic data. Zoological Journal of the Linnean Society, 184:528–583, doi: 10.1093/zoolinnean/zly007.

**Phylogenetic tree (Newick format)**

((HGDP00958.0:0.422251,(((((((((((((((((((((HG01817.1:0.2002979661,HG02087.1:0.2007151105):0.048652,HG02399.1:0.2073917862):0.029431,((HGDP00718.0:0.1578400592,HGDP00713.0:0.1318909135):0.074232,(HGDP00718.1:0.1554622504,HGDP00713.1:0.1609571129):0.072581):0.057169):0.028639,((HG01841.0:0.2077454156,HG00729.0:0.2059125002):0.060053,((NA19006.0:0.1961505335,HG02407.0:0.2091450208):0.037287,(HG01846.1:0.1901909791,HGDP01239.1:0.1978952264):0.048178):0.020907):0.023515):0.019282,((HG02380.0:0.1708942555,HG02156.0:0.1541601148):0.093329,(((HG00879.0:0.2163425153,HG02121.1:0.1813819750):0.060113,(NA18986.1:0.2042747693,(NA19007.0:0.1682311165,HG00759.1:0.1899814312):0.044434):0.027421):0.018814,(NA18979.1:0.2078105095,NA18945.1:0.1884064529):0.059105):0.029030):0.018348):0.011724,((((HG02410.1:0.1767261381,HGDP00719.0:0.2258819267):0.043075,HG02087.0:0.2075341117):0.036442,((((HG02182.0:0.1606006807,HG02380.1:0.1724256835):0.049788,HG02188.1:0.1981306722):0.020137,HG01801.0:0.2089500508):0.053569,(HG02409.0:0.1962820834,HGDP01179.1:0.2081326668):0.052569):0.021932):0.024526,((((HG02078.0:0.1717256451,HG02081.0:0.2014779888):0.075569,(HG00437.0:0.1908696631,HG01595.1:0.2015285511):0.046943):0.026495,((HG02070.1:0.2051526575,HG02521.0:0.1995701994):0.037496,HG02017.0:0.2139581635):0.039288):0.023328,((HG00410.1:0.2095976799,HG01813.0:0.1971436803):0.054050,((HG01046.1:0.1970698845,HG00543.0:0.1877298275):0.050974,((HG02070.0:0.1873378293,HG01871.1:0.1930926503):0.045858,(HGDP00717.1:0.2049721796,HG00693.0:0.2060308966):0.066271):0.014743):0.023997):0.033911):0.011251):0.014295):0.010550,(((((HG02127.0:0.2050117485,HG02367.1:0.1732178589):0.044943,(HG01868.1:0.1907027849,HG02187.0:0.2281464404):0.037392):0.026698,(HGDP01239.0:0.2063155256,(HG00407.1:0.2044832998,HG02075.1:0.1886251774):0.039642):0.033613):0.022616,(((HG02016.1:0.1979847058,HG02113.0:0.1949221076):0.048041,HGDP00715.0:0.2208321707):0.022697,HG01849.1:0.247622):0.021220):0.018070,((HG02057.1:0.2401621389,(HG02127.1:0.2181262089,HG02085.1:0.1859301326):0.047399):0.027027,((HG01861.1:0.1955206555,HG02367.0:0.1853640951):0.047220,(HGDP00720.0:0.2026035002,HGDP00715.1:0.2245625934):0.051902):0.034158):0.033304):0.019876):0.009665,((((HGDP01349.1:0.2103401043,HG00536.1:0.2005727312):0.049503,(HGDP01188.0:0.243521,HGDP01207.0:0.233164):0.021155):0.021598,((((HGDP01250.0:0.1967182274,NA18528.0:0.1964835311):0.050898,(HG00533.0:0.1813305371,HGDP01236.0:0.1875937235):0.050667):0.019025,(HGDP01342.0:0.1816905045,HGDP01339.0:0.2086538613):0.061266):0.020887,((HGDP01353.0:0.2051888597,NA18595.0:0.1813935033):0.055500,(NA18997.0:0.2038735100,HGDP01228.0:0.1932903910):0.039525):0.032906):0.013312):0.019501,((((NA18557.1:0.1960226817,HG02086.0:0.1995696290):0.064487,(((HG00864.1:0.1750679908,HG01844.1:0.2102090520):0.050614,HGDP00714.1:0.2234268236):0.020144,(HG02061.0:0.1889410770,NA18748.0:0.1875380810):0.042341):0.029068):0.020083,(((NA18579.1:0.1851814311,HG02152.1:0.1913064477):0.046533,(HGDP01223.1:0.1844340529,NA18563.0:0.1797007018):0.054021):0.026961,((NA19070.0:0.2121940621,NA18959.0:0.1744232017):0.044003,((NA18987.0:0.1772975735,HG02385.0:0.1828582543):0.058506,HGDP01251.0:0.2174584194):0.020015):0.030619):0.016126):0.014198,((HG02035.1:0.1955151963,HG02020.1:0.1782171990):0.058294,(((NA18599.1:0.1961879222,HG01805.0:0.1927982497):0.044172,HG00531.1:0.1990433560):0.030353,(HG00406.0:0.1780679523,NA18960.0:0.1935524497):0.046711):0.014873):0.026629):0.021335):0.015253):0.007242,(((((HG00410.0:0.1981071546,HG00663.1:0.1876346424):0.053394,(HG00478.1:0.1881618629,HG01872.1:0.1991685583):0.060982):0.017312,(((HGDP01186.1:0.1756809071,HG01845.1:0.2204057217):0.043776,HG01874.1:0.2061510096):0.029757,(HG00864.0:0.1906135473,HG02374.1:0.2009942153):0.040455):0.024411):0.037214,(((((NA18539.1:0.1845230652,NA18606.0:0.1944446949):0.050402,(NA18606.1:0.1999471307,HG02155.0:0.1959863497):0.030798):0.026841,((NA19010.1:0.1908392205,HG01800.1:0.1791136807):0.037833,NA19054.1:0.2185789575):0.035602):0.021651,((NA19080.0:0.2113267914,HG00559.0:0.1922134436):0.058667,(HG00717.1:0.1820010867,NA19091.1:0.2086976636):0.053230):0.029844):0.021950,((((HG02179.1:0.1876369723,HG02395.0:0.1996047872):0.063394,NA19077.0:0.24056):0.036287,((HGDP01192.1:0.1785418108,HGDP00717.0:0.2083679281):0.049964,(HG02032.0:0.1833402955,HG01599.0:0.1814862608):0.069871):0.021635):0.018837,((HG00982.0:0.1997534463,HGDP01338.1:0.1902391555):0.038946,HG01867.1:0.2284355474):0.055187):0.020881):0.011891):0.010420,((((((HG02165.0:0.1847220208,HGDP01332.1:0.1858486173):0.034758,(HG02250.1:0.1744299546,HG01853.0:0.1953704588):0.050314):0.027667,(HGDP01096.1:0.2128387174,HG00525.0:0.1691003748):0.059373):0.021716,(HG02375.0:0.1739841295,HG01046.0:0.1921138511):0.078688):0.021487,(NA18615.1:0.1933511105,HG01599.1:0.1868062753):0.090222):0.012545,(((HG00844.1:0.2005645997,HG02140.0:0.1874085392):0.054350,HG01596.1:0.2098450173):0.030099,((HG02383.1:0.2094329587,HG02384.0:0.233821):0.024795,HG02384.1:0.218525):0.026392):0.022996):0.024118):0.010199):0.007399,(((((((HG02379.0:0.1877860408,HG00409.1:0.1852295940):0.053827,HG02082.0:0.238704):0.020211,(((HG01858.0:0.1790678023,HG01598.0:0.2157516244):0.041149,HG02134.1:0.2051945142):0.019364,(HG00421.1:0.1839633506,HG02178.0:0.1947429259):0.034988):0.028542):0.016938,(NA18635.1:0.279264,(HG01867.0:0.1988526513,HGDP01326.0:0.1781613642):0.046577):0.034594):0.024524,((((HG00631.0:0.2087180570,HG02386.0:0.1697437624):0.047656,(HG01842.1:0.2284046005,HGDP01187.0:0.218322):0.030373):0.020851,((HG01809.1:0.1989159442,HG01847.0:0.1927795966):0.052889,(HG01797.0:0.1909462292,HG02025.1:0.1900243648):0.046674):0.039413):0.014826,((((NA18565.0:0.1844531734,HGDP01098.1:0.1718064797):0.046509,HG01862.0:0.2076407787):0.017621,(HG01855.1:0.2008460327,NA18988.1:0.1846891907):0.044096):0.028136,(HG01031.0:0.1946927513,NA18559.1:0.261638):0.040873):0.021734):0.016515):0.010069,(((((HGDP01250.1:0.2059709743,(NA18525.0:0.1624629936,HG00656.0:0.2049637762):0.034954):0.028752,HG01812.0:0.2114920080):0.030856,(NA18995.0:0.235536,HG00614.0:0.2180053053):0.024716):0.029224,((NA18637.0:0.2198919954,(HG02020.0:0.1809365358,NA18543.1:0.1827361880):0.042979):0.034868,(NA18648.1:0.1990479296,NA18622.0:0.1798935032):0.051413):0.028621):0.023713,(((((NA18644.0:0.1802425088,NA18612.1:0.1906894000):0.041595,(HG01864.0:0.1967892726,HG00851.0:0.1821418896):0.052726):0.033724,HGDP01246.0:0.226396):0.023834,(((HG02049.1:0.1990001675,HG02019.1:0.1768838218):0.066185,HG01857.1:0.2148394663):0.024644,((HG02084.1:0.2019268528,HGDP01350.1:0.2023105798):0.036740,HGDP01100.1:0.1892243777):0.042243):0.020209):0.018246,((NA18639.1:0.1962010643,HGDP01187.1:0.1937347214):0.064383,((HG00557.0:0.222479,(NA18542.0:0.1958490884,HGDP01186.0:0.1868024154):0.050163):0.024629,(NA18542.1:0.2064963510,(HG01596.0:0.1820888321,HG00717.0:0.1961311588):0.038671):0.036831):0.017569):0.013856):0.020377):0.015513):0.006865,((HG02078.1:0.255114,((HG02188.0:0.1695597090,HG01806.1:0.1734092209):0.042326,HG01805.1:0.1987874421):0.034613):0.033961,((HG02389.1:0.260112,(NA18544.1:0.1959195709,HGDP01335.1:0.1883892366):0.058337):0.022570,((HG02390.1:0.1665645464,HG00629.1:0.2015432031):0.039258,HG02521.1:0.2129779220):0.036105):0.023926):0.028412):0.008703):0.005714,((((((((((HG01842.0:0.1694361649,HG02067.0:0.1685174565):0.060578,(HG00982.1:0.1885953271,NA18974.0:0.1784348659):0.044584):0.008880,((HG01808.1:0.1928852086,HG01878.0:0.1875880192):0.057384,(HG01869.1:0.1802830123,HG01840.1:0.1780893990):0.042520):0.018799):0.026464,(HG02390.0:0.1941807788,(HG00565.1:0.1995288783,HG01870.0:0.1720207834):0.037444):0.045275):0.019428,(((NA18633.1:0.1731340652,HGDP01354.1:0.1953053126):0.056406,HG00479.0:0.228864):0.028321,((NA18978.1:0.1899977631,HG01799.0:0.1794231597):0.072641,((HG02076.0:0.1743123649,HG01866.1:0.1943431842):0.039800,HG00422.0:0.250853):0.026361):0.022987):0.012884):0.018200,(((((HG00663.0:0.1954987602,HG00683.1:0.1776545074):0.039244,HG00560.0:0.2020123993):0.032727,(HG00701.1:0.1779758711,NA18550.0:0.1801541625):0.039678):0.027124,((((HGDP01331.0:0.1900513604,HG00472.0:0.2004542677):0.051527,(HGDP01198.1:0.1793609680,HGDP01195.1:0.1976111994):0.050556):0.020478,HG00565.0:0.230025):0.026348,((NA18603.0:0.1712788718,HG00690.0:0.1859613100):0.045963,NA18549.0:0.1892233789):0.040762):0.021166):0.016465,((NA18567.0:0.2078509196,(NA18560.0:0.1704322896,NA18617.1:0.1766889771):0.035780):0.031539,((HG00622.1:0.1936804370,(HG02513.0:0.1950610890,HGDP01100.0:0.1871335377):0.046584):0.029788,(HG00626.1:0.1754491172,HG02181.0:0.1942084279):0.061914):0.030675):0.020656):0.013978):0.011576,(((((HG02075.0:0.1604833567,HG02130.1:0.1912521919):0.070679,((HG02382.1:0.1402864258,HG02375.1:0.2037933663):0.032109,HG02407.1:0.1868491972):0.042127):0.023289,HG01815.0:0.2284994820):0.026226,((HG02130.0:0.2079585296,(HGDP01180.0:0.1920059164,HG00705.0:0.1797028057):0.049437):0.024799,(((NA18644.1:0.1897522277,NA18593.1:0.1706731346):0.045357,NA18632.1:0.2176956404):0.023048,(HG02397.1:0.1731627165,HG02513.1:0.1977651272):0.049633):0.021139):0.016523):0.019972,(((HG01863.1:0.1944308947,HG00524.1:0.1887912202):0.046361,((HG02086.1:0.224952,HG02025.0:0.2115211649):0.025427,(HG02113.1:0.1953135917,HG02134.0:0.1807861969):0.054395):0.021893):0.027111,(((((NA18974.1:0.1838783624,NA18976.0:0.1933246908):0.050648,NA18749.0:0.2215614056):0.019434,(NA18605.1:0.1758669768,NA18605.0:0.1883151870):0.046175):0.032116,((NA18611.0:0.1890710446,HG02408.0:0.1955733243):0.035697,(HG00436.1:0.1821575056,HGDP01193.1:0.1992716743):0.058046):0.023796):0.017801,(HG02351.0:0.236988,(HG02136.1:0.1663893170,HG02396.0:0.2005669649):0.057377):0.027853):0.014459):0.018491):0.015030):0.007167,(((((((HG01809.0:0.1884950415,NA18555.0:0.1793693888):0.040829,NA19063.1:0.2111731316):0.033906,(HG01865.1:0.1759498521,NA18640.1:0.1921008936):0.057333):0.020703,((HG02142.0:0.2396310631,NA18634.0:0.240611):0.032049,(HGDP01341.0:0.2269759994,(HG01600.0:0.1857436461,HG00704.0:0.2095229981):0.041384):0.036476):0.022944):0.020079,((HG02032.1:0.1889001914,(NA18942.1:0.1852183818,NA18621.1:0.2000369124):0.041309):0.030222,((HG01878.1:0.1792156539,HG02026.0:0.1815788486):0.040063,HG01598.1:0.2201421030):0.036824):0.038764):0.015873,(((NA18577.1:0.216356,(NA19083.0:0.1955694229,NA18959.1:0.1818464922):0.052072):0.025815,((HG02164.1:0.1982269645,HG02397.0:0.1644552597):0.067479,(NA19081.1:0.1862592416,NA18563.1:0.1915788124):0.046880):0.015094):0.024988,(((HGDP01225.1:0.1783426208,NA18596.0:0.1931124553):0.039959,(HGDP01104.0:0.1682058967,HG01595.0:0.1948696767):0.049153):0.031436,((HGDP01319.1:0.1795383055,HG01843.1:0.2032229384):0.041610,NA18949.0:0.2240257846):0.030216):0.017566):0.018447):0.007431,(((HG00556.0:0.1767817717,HGDP01180.1:0.1919032759):0.047540,((HG02064.0:0.2001740477,HG02153.1:0.1947617959):0.037695,(HGDP01354.0:0.2098615526,HG02186.0:0.1847121841):0.041863):0.026272):0.028864,(((NA18603.1:0.1741406856,NA18571.0:0.1874719465):0.070174,(NA19006.1:0.2092816441,HG00619.1:0.212971):0.041801):0.020059,(HG00608.1:0.231485,(NA18973.0:0.1796338417,NA18745.1:0.2002470283):0.059239):0.030052):0.014914):0.017150):0.017174):0.005366,((((((HG01840.0:0.242099,(HG01028.1:0.1853352304,HG00613.1:0.1903562948):0.040400):0.042222,(HG02137.0:0.221611,(HGDP01232.0:0.1745265704,HG00599.0:0.1867724627):0.056334):0.04402):0.015888,((((NA18630.0:0.1832191044,HG00867.1:0.1806706258):0.040586,HG00500.0:0.2068970472):0.035539,((NA18617.0:0.1874575103,NA19082.0:0.1933902954):0.038488,(HG00620.0:0.1854260276,HG00451.0:0.1944063783):0.051871):0.022503):0.020212,((HG02048.0:0.223614,(HG01859.0:0.1672355268,HG01851.1:0.1928103312):0.048197):0.015080,(HG02184.0:0.1709653254,HG00422.1:0.1849011330):0.064256):0.019999):0.008393):0.013353,((HGDP01099.1:0.1945956193,HG02040.1:0.1960380798):0.062784,((HG02391.1:0.1844685702,HG01794.1:0.1890150992):0.048419,((HG01795.1:0.1361417258,HG02179.0:0.1123313378):0.090664,HGDP01317.0:0.1770223796):0.041486):0.031715):0.022841):0.019164,(((((HG00705.1:0.1650577986,HG00654.1:0.1802695691):0.040283,HG01843.0:0.2197947374):0.030048,((HG01794.0:0.1792066730,HG01813.1:0.1710069081):0.050604,HG01870.1:0.1999093099):0.024384):0.019965,((HG02402.0:0.1763733047,HG01847.1:0.1859064841):0.037581,HG01815.1:0.2179069168):0.046445):0.021000,((((HG02088.0:0.1505183759,HG00613.0:0.1797897419):0.046691,(HG02072.1:0.1684881944,HG02122.1:0.1827213197):0.057690):0.019610,((HG01796.0:0.1742910210,HGDP01192.0:0.1879402060):0.035989,NA18609.1:0.2212191106):0.022546):0.014693,HG01872.0:0.241598):0.028445):0.014691):0.011225,((((((HG00566.0:0.1898583637,NA18591.1:0.1685112952):0.053998,((HG00479.1:0.1750841429,NA18560.1:0.2055860456):0.046682,(HGDP01182.0:0.1798765597,HGDP01337.0:0.1750799723):0.049465):0.021525):0.021962,((HGDP01097.0:0.248844,(NA18618.0:0.2097269050,HGDP01183.1:0.1659974905):0.047236):0.016098,HG01807.0:0.256672):0.022098):0.020876,((NA18981.0:0.245173,(HG00623.1:0.1955848665,HG02185.1:0.1924415722):0.046265):0.035892,(((HG00463.0:0.1789153803,HG02061.1:0.1854800154):0.034499,HG00583.1:0.2222347149):0.029248,(HG02353.0:0.1868924700,HG02364.1:0.1885297215):0.049932):0.018273):0.022469):0.011872,(((HGDP01190.0:0.1926122373,HGDP01197.1:0.2042655441):0.054815,((HG02085.0:0.1796244421,NA19055.1:0.1995811479):0.043996,(HG02031.1:0.2133829524,NA18988.0:0.1757667741):0.044132):0.023850):0.024166,(((HG01597.0:0.23802,NA18573.0:0.234871):0.026520,((NA18990.1:0.1967667839,HGDP01224.1:0.1860107072):0.034792,HG00675.1:0.2025610246):0.033599):0.016854,((HG02395.1:0.1719578890,HG01795.0:0.1804866587):0.069803,NA18983.1:0.248485):0.022879):0.011104):0.015631):0.012661,((((HG00451.1:0.1827986997,HG00566.1:0.1933589759):0.048763,(HG00595.0:0.1973729170,HG01852.0:0.1900645723):0.050249):0.034482,(HG00707.1:0.24532,((NA18582.0:0.2029549422,NA18592.0:0.1851484752):0.044766,(HG02402.1:0.1969344663,NA18636.0:0.1687058270):0.042115):0.017763):0.017238):0.024016,((NA18638.1:0.1926387042,NA18537.1:0.1868142197):0.046030,(HGDP01235.0:0.1874322050,HG00654.0:0.1881382395):0.052661):0.029470):0.023405):0.007356):0.008890):0.004286,((((((((((NA18962.1:0.226469,NA18999.0:0.1761966045):0.055804,(NA19067.0:0.1692541875,NA19077.1:0.1936622479):0.080164):0.014622,(((NA18941.1:0.1772449093,HG00766.1:0.1960811160):0.055388,((NA18965.1:0.1676650583,NA19083.1:0.2061688911):0.049609,NA19062.1:0.1957143472):0.037398):0.019040,((NA18968.1:0.1955278484,NA18940.0:0.1853608933):0.043389,NA18991.0:0.2020197581):0.035965):0.018718):0.014301,((((((NA19011.1:0.2090829259,NA19088.1:0.1811290203):0.043320,NA18941.0:0.1831726122):0.035935,(NA18975.1:0.2022533889,NA19087.0:0.1827206894):0.046696):0.021192,(NA18956.1:0.1860193322,NA19059.0:0.1772830827):0.059659):0.014644,((NA18992.1:0.24285,(HGDP01238.1:0.2150527122,NA19060.1:0.1645599319):0.036449):0.017183,(NA18968.0:0.1903331028,NA19003.0:0.1948033967):0.054262):0.027479):0.020083,((NA19012.1:0.266229,(NA19074.1:0.226448,(HGDP01222.0:0.2004530438,NA19078.1:0.1988436807):0.047547):0.032086):0.018490,((NA19072.0:0.253751,(HG01798.1:0.1579018537,NA19075.1:0.1842137219):0.047768):0.018242,NA18939.1:0.227522):0.027404):0.013977):0.016916):0.011713,((NA18624.1:0.218761,(NA18956.0:0.1774711141,NA18998.1:0.1950814691):0.056602):0.033486,((NA19002.0:0.1862692170,NA19088.0:0.1932888048):0.054427,((HGDP01241.1:0.1914438447,NA18994.1:0.1870262485):0.042455,NA18978.0:0.2130987310):0.022911):0.021655):0.030081):0.009774,((((HGDP01213.1:0.1803750430,HGDP01206.0:0.1957569683):0.033056,NA19086.0:0.1914381415):0.017001,HGDP01350.0:0.2285740405):0.026993,(((NA18635.0:0.1789238813,NA18944.1:0.1854435890):0.045725,NA18973.1:0.1932233091):0.021737,(NA19081.0:0.218374,(NA18957.0:0.1923701487,NA19010.0:0.1896465716):0.052389):0.020324):0.017368):0.025666):0.014774,((((HGDP01227.0:0.2234451329,(HGDP01181.0:0.1886413518,HG00608.0:0.1891149218):0.037254):0.027599,(NA18622.1:0.239305,HGDP01245.0:0.248484):0.029328):0.020798,((HG00584.1:0.1906306711,HGDP01331.1:0.1735456330):0.049950,(HG02026.1:0.2114951515,HGDP01231.1:0.1915105709):0.039487):0.041182):0.019806,((HG00458.1:0.255477,HG00473.1:0.2113424216):0.022017,HG00406.1:0.238467):0.033456):0.018700):0.008264,(((((HG01858.1:0.1922703458,HG02408.1:0.1985917844):0.057595,(NA18643.0:0.1790122160,HG01852.1:0.1819815830):0.052058):0.021095,NA18546.1:0.238022):0.017933,(((HG02184.1:0.1812718630,HG02373.0:0.1781218866):0.056189,(HG01860.0:0.1846307161,HG02121.0:0.1937717874):0.045208):0.018586,((HGDP01323.0:0.1653083237,HGDP01324.1:0.1392782083):0.044837,(HGDP01323.1:0.1096853878,HGDP01324.0:0.1380150249):0.068470):0.060846):0.030378):0.022513,(((((HG00728.0:0.1941887824,HG00557.1:0.1821508201):0.046658,HGDP01237.0:0.2163576799):0.025761,((HG00449.1:0.1871412358,NA18626.1:0.1819991138):0.049302,NA18977.1:0.248065):0.019409):0.025245,((((NA18609.0:0.1841255678,HG00464.0:0.1963160257):0.041840,(NA18643.1:0.1910940553,NA18531.0:0.1873007763):0.040833):0.025039,(HGDP01217.1:0.1733646600,HGDP01226.1:0.1997822816):0.057691):0.025375,((NA18531.1:0.2058792947,HGDP01214.1:0.233659):0.033931,((NA18980.0:0.2034195836,NA19059.1:0.1858733996):0.040474,HGDP01209.1:0.2239870776):0.027133):0.023494):0.016401):0.011560,(((HGDP01232.1:0.1916644871,NA18950.0:0.1784147393):0.045697,(NA18951.0:0.254852,(NA18977.0:0.1852923216,HGDP01207.1:0.1977800167):0.044265):0.025122):0.024551,((HG00684.0:0.1859683343,HG02512.1:0.1938892580):0.039879,(NA18553.1:0.1982400186,HGDP01215.1:0.2063664019):0.053605):0.037391):0.025009):0.015417):0.008606):0.008217,(((((((HG00581.1:0.1716064127,HG00607.1:0.1618368056):0.068790,(HGDP01338.0:0.2002158348,HGDP01181.1:0.1734729836):0.051854):0.023968,((HGDP01327.0:0.1872299002,NA18627.0:0.1780139053):0.044231,(HG00632.1:0.1791327058,HGDP01184.0:0.2072229370):0.050682):0.022883):0.019079,(((NA18625.0:0.1714741479,NA18634.1:0.1872210257):0.058883,(HG00656.1:0.1943412103,HGDP01332.0:0.1626371636):0.043575):0.020987,((HG02399.0:0.1722919046,HG01862.1:0.1952322894):0.050706,(HG00759.0:0.2116364859,HG00956.1:0.1677058046):0.048169):0.029633):0.018199):0.011920,((HG00881.0:0.1583342779,HG00879.1:0.1897819727):0.065114,(NA18749.1:0.1985104906,HG01802.1:0.1788944644):0.068121):0.026787):0.010002,(((((((HG00672.1:0.1752410083,HGDP01348.1:0.1852464934):0.035683,(HG00653.0:0.1607384812,HG00671.0:0.1643046088):0.066190):0.017070,HG00651.1:0.2091364341):0.028085,((NA18550.1:0.1940445208,HG00628.1:0.1862767616):0.037908,(NA18597.0:0.1905568348,HGDP01097.1:0.1788232083):0.033454):0.032122):0.022696,((HG00589.1:0.1942189224,NA18562.1:0.1743490810):0.037738,(HGDP01185.1:0.2041915477,HGDP01339.1:0.1878057404):0.055273):0.031986):0.019226,(((HG00628.0:0.1764814021,NA18631.0:0.2058408446):0.041626,NA18558.1:0.2114994489):0.029767,(HGDP01102.1:0.1721614422,HGDP01355.1:0.2031656834):0.059349):0.030956):0.014908,((((HG00592.0:0.1920931851,NA18548.1:0.1883785170):0.044401,(HG01810.1:0.1877885859,HG00452.0:0.2040126816):0.054646):0.027414,(((HGDP01190.1:0.1855514685,HGDP01101.0:0.1928361882):0.044936,NA18574.0:0.243946):0.013740,HG02029.0:0.246476):0.016411):0.020782,((NA18541.0:0.1702393533,NA18946.0:0.2042419505):0.042984,(HG00629.0:0.1957998863,HG00419.0:0.1882547110):0.054746):0.034770):0.016016):0.013254):0.011291,((((((NA18545.1:0.1918655502,NA18953.1:0.1813086863):0.041217,(HGDP01197.0:0.225663,(HG00662.1:0.1767204422,HG00698.1:0.1784882201):0.054456):0.029544):0.023999,((HG02166.1:0.1821174529,HG02522.1:0.1602796671):0.069442,(NA18546.0:0.1692155396,NA18620.1:0.2011311329):0.048832):0.026669):0.018735,((((NA18641.0:0.255469,HGDP01333.1:0.1881170354):0.020540,(HGDP01330.1:0.1744534030,HGDP01327.1:0.1784730939):0.040530):0.025148,HGDP01244.0:0.231764):0.017897,(((NA18954.0:0.212683,NA18970.0:0.1992725936):0.031792,(NA18570.0:0.1946824198,NA19068.1:0.1803244489):0.040922):0.023537,((NA19007.1:0.1846252356,NA18943.0:0.1802956318):0.054618,(NA19058.1:0.1976655446,NA19012.0:0.1579272159):0.044896):0.027648):0.015535):0.016650):0.018146,(((((HGDP01247.0:0.1874874929,HGDP01194.1:0.2058802629):0.054706,((NA18530.1:0.1928213094,HG00596.0:0.1892288031):0.054375,(NA19091.0:0.2119323781,HGDP01196.0:0.1769776329):0.060142):0.016918):0.018550,((HG00537.1:0.1749964196,HG00534.0:0.1569304080):0.067575,(NA19068.0:0.1869879549,HG00437.1:0.1913466247):0.049255):0.021137):0.024401,((HG00634.1:0.239176,NA18628.1:0.2164303927):0.031057,NA18526.0:0.213718):0.026708):0.014671,((HG00473.0:0.1934608256,NA18747.0:0.1863759180):0.050915,((NA18613.1:0.1853779307,NA18558.0:0.2071392281):0.052774,((NA18630.1:0.1811629574,HGDP01335.0:0.2027656478):0.045807,HG00458.0:0.1926239639):0.033709):0.015018):0.031049):0.013544):0.008784,(((((((HGDP01336.1:0.1775151939,HGDP01189.0:0.1848919991):0.032997,NA18942.0:0.236706):0.025295,(HGDP01329.0:0.1835884048,HGDP01334.1:0.1757359164):0.055691):0.022459,((HGDP01328.0:0.234112,NA19076.0:0.218741):0.025182,(HGDP00714.0:0.2076448212,HG02351.1:0.1743416505):0.052752):0.017269):0.014650,((((HG02154.1:0.1720622399,HG02154.0:0.1619595981):0.062258,HG02364.0:0.2070631886):0.043216,(NA18614.1:0.2198763645,(NA18621.0:0.1784047430,NA18602.0:0.1897449415):0.042043):0.031233):0.023266,((HG01808.0:0.1915751280,HG00536.0:0.1782578413):0.043484,(NA18619.0:0.1861209875,NA18573.1:0.1971100818):0.045414):0.033143):0.024446):0.018721,((((NA18591.0:0.1849791025,NA18597.1:0.1842561165):0.052818,((NA18564.0:0.1880760803,HGDP01238.0:0.1704227945):0.035539,NA18574.1:0.2086039118):0.034970):0.018362,((NA18951.1:0.1866159950,NA18997.1:0.2055399883):0.037615,NA18629.0:0.1925019556):0.037610):0.023715,(((HGDP01193.0:0.2019834383,(HG02373.1:0.1801425825,HGDP01195.0:0.1719888882):0.052438):0.025136,(HGDP01185.0:0.1836685670,HG02058.0:0.1878252043):0.051887):0.024256,(HG02389.0:0.2011890622,HGDP01189.1:0.1877689625):0.057159):0.019754):0.011172):0.009909,(((NA19004.1:0.230436,(NA18983.0:0.2067428871,(HGDP01330.0:0.1937822528,HGDP01208.0:0.2103961947):0.038820):0.032930):0.023192,((HG00581.0:0.1777508102,HG00580.1:0.1686004360):0.050115,(NA18952.0:0.1865501664,NA18541.1:0.1692839494):0.041122):0.027538):0.025187,((NA18994.0:0.219019,HG00513.0:0.237794):0.029993,(((HG00583.0:0.2109132263,HG01844.0:0.1527595173):0.033895,HG02047.0:0.2100269207):0.033470,((HG00596.1:0.1686390809,NA18639.0:0.1910082736):0.041835,NA18572.0:0.1879500418):0.023847):0.029475):0.017226):0.016435):0.007112):0.007698):0.006538):0.011398,((((((((HG02016.0:0.23519,(HGDP01336.0:0.1859403480,NA19072.1:0.1826254614):0.037618):0.037537,(((NA18616.1:0.1596775111,HGDP00711.0:0.2221823452):0.045148,(HG02073.0:0.1976137555,HG01851.0:0.1876127495):0.064513):0.017567,(HG02151.0:0.1478668190,HG01810.0:0.1607632904):0.107332):0.018568):0.011114,(((HG01863.0:0.1719930234,HG02401.0:0.1894541541):0.045168,NA18748.1:0.1939650654):0.023499,(HG02398.0:0.1901830270,HG01804.0:0.1869761265):0.049127):0.033801):0.018017,((HG02386.1:0.1952328654,HG02355.1:0.1849186021):0.062903,(((HG01812.1:0.1604407418,HG01800.0:0.1771247894):0.053531,HG02142.1:0.2295954959):0.020872,(HG01796.1:0.1848241183,HG02401.1:0.1732437258):0.050155):0.026122):0.021902):0.019541,(((HG02139.1:0.2102102421,(HG00707.0:0.1796902991,HG02023.1:0.1884252827):0.040296):0.046428,(((HG01811.0:0.1821781194,HG00593.1:0.1816663503):0.044054,(HG00543.1:0.1791188031,NA18602.1:0.1609765225):0.041243):0.024141,HG02031.0:0.252847):0.014386):0.017917,(((HG01816.0:0.1848260167,HG01811.1:0.1776330921):0.054369,HG02374.0:0.1932424319):0.036639,(NA18637.1:0.212208,(HG02047.1:0.1932447621,HG02371.1:0.1714740769):0.053499):0.023110):0.012234):0.019101):0.009936,((((HG02353.1:0.1468847226,HG02379.1:0.1681275015):0.081352,(HGDP01098.0:0.1799538831,HG02250.0:0.1857461037):0.032539):0.043842,((HG02023.0:0.2279597278,(HG00533.1:0.1848289408,HG02140.1:0.1824491617):0.047804):0.044494,(((HG02164.0:0.2002295709,HGDP01345.1:0.1822087852):0.050825,(HGDP01103.0:0.1831707059,HG00530.1:0.1794662305):0.047406):0.021929,(HG00625.0:0.248694,(HG00419.1:0.2216747963,(HG00403.0:0.1722494444,NA18557.0:0.1994476950):0.040570):0.025338):0.027352):0.015603):0.016250):0.017728,((((HG01600.1:0.2046270073,NA18593.0:0.1852631038):0.042790,HG02029.1:0.2241987212):0.034247,(HG02155.1:0.1977998884,(HG02084.0:0.1610167408,HG02138.1:0.2236772562):0.037173):0.024732):0.030058,(HG02396.1:0.1793229111,NA18969.1:0.2073628768):0.064862):0.024309):0.015359):0.011086,((((((HGDP01216.0:0.1896970857,NA18939.0:0.1934719954):0.051652,(NA18525.1:0.233378,(NA18948.0:0.1952656308,HGDP01249.1:0.1915461081):0.040866):0.022561):0.027278,(NA18616.0:0.220991,(NA18962.0:0.2229920214,NA18582.1:0.254686):0.030204):0.030558):0.015403,(((HG00472.1:0.1695826568,NA19056.0:0.1988053552):0.043766,NA18990.0:0.2071678189):0.037393,((NA19070.1:0.1867878930,NA19084.1:0.1710191207):0.045325,(NA18940.1:0.1953601677,NA19086.1:0.1853548222):0.038794):0.026907):0.027639):0.014657,((((NA18572.1:0.1879020177,HG02406.1:0.1957955284):0.055891,((HG00657.1:0.1702655767,HG02128.1:0.1910346906):0.036349,HG01807.1:0.1958741044):0.021803):0.025291,(((HG00589.0:0.1735321448,HG00598.0:0.1963189444):0.044946,(HG00595.1:0.1944760605,HG00692.1:0.1686160836):0.043005):0.025730,(NA18954.1:0.1858196240,NA18645.0:0.1917079599):0.057861):0.025813):0.014620,((HG00674.0:0.1765563437,HG00631.1:0.1672397833):0.060057,(HG02186.1:0.236854,(HGDP01334.0:0.1878279616,HG00513.1:0.1897626094):0.059055):0.019474):0.028935):0.019630):0.011171,((((((HG00475.0:0.1816635935,HGDP01244.1:0.1924440107):0.043985,(NA18971.1:0.1875751951,HG01817.0:0.2004887467):0.052503):0.020504,((HG02081.1:0.1997632236,(HG01865.0:0.1863612818,HG00436.0:0.1889163994):0.038123):0.032694,((NA18971.0:0.1697217113,NA18636.1:0.2058802573):0.035382,NA18943.1:0.2059452716):0.030416):0.028772):0.013033,((NA18549.1:0.1855216615,NA18992.0:0.1959792787):0.047891,(NA18981.1:0.1732905807,NA18989.0:0.1894796853):0.050872):0.032847):0.012218,((HG02057.0:0.2008950031,HG00580.0:0.1892229892):0.057888,(NA19075.0:0.1695700461,HG02392.1:0.1998149683):0.050329):0.036147):0.017504,(((((HG02190.1:0.1963081046,HG00442.0:0.1744971794):0.048050,(HG00674.1:0.2241364075,HGDP00711.1:0.1791145954):0.075588):0.031011,(HG00476.0:0.256141,((NA19090.1:0.2123914508,NA18957.1:0.1794683657):0.049166,HG02028.1:0.2281376326):0.025853):0.036599):0.014216,(((NA18999.1:0.1884086959,NA18947.1:0.1776570470):0.043150,(HG00404.0:0.2127234643,(NA18547.0:0.1846790481,HG00457.1:0.1774688773):0.044435):0.033423):0.019144,((((NA19009.0:0.1927136814,NA18969.0:0.1707463611):0.049807,NA18993.1:0.1984894509):0.032344,(NA18944.0:0.1930701107,(HG00593.0:0.2025772481,NA18946.1:0.1726410425):0.045230):0.018137):0.028402,NA18953.0:0.2128836615):0.028613):0.016151):0.010305,(((HG00524.0:0.1677760482,HG00446.0:0.1930919446):0.054147,(HG02360.0:0.1870254796,HG00442.1:0.1904686721):0.061369):0.026056,(HGDP01333.0:0.234404,NA18562.0:0.2006106043):0.036745):0.024145):0.009536):0.013757):0.008193):0.007608,((((((HG02040.0:0.1779992215,HGDP00712.0:0.1833239124):0.048763,((HG02382.0:0.1997377146,(HG01597.1:0.1716386653,HG01853.1:0.1953148109):0.049474):0.026116,(NA18595.1:0.1822215730,HG00626.0:0.1792964148):0.039619):0.017994):0.023530,((HG02136.0:0.2070383955,NA19057.0:0.1884016682):0.056606,(NA18532.1:0.1897995260,NA18646.0:0.1922677908):0.048531):0.026031):0.015174,(((HG02076.1:0.1843958642,NA18545.0:0.1856133073):0.037420,(HG00766.0:0.2176201049,NA18632.0:0.2000392162):0.028428):0.041106,(HG00584.0:0.2268751320,(HGDP00712.1:0.2026361093,HGDP01328.1:0.1729470065):0.041341):0.030904):0.020838):0.022881,((((NA18608.0:0.1978258247,HG02058.1:0.1953325944):0.058050,((NA19079.1:0.2058971925,NA18566.1:0.1917303406):0.046740,(NA18571.1:0.1633838357,HG00525.1:0.1888442708):0.045507):0.019453):0.022075,((HG00556.1:0.1825291928,HG00701.0:0.1920738926):0.055223,(NA19065.0:0.240579,(NA18757.0:0.1871103618,HG00443.1:0.1953568178):0.047355):0.018036):0.028395):0.018433,((HG00683.0:0.241029,NA18965.0:0.231281):0.024483,((HGDP01347.1:0.2166411837,HG00590.0:0.1917247458):0.054869,NA18552.1:0.234382):0.029402):0.025218):0.011854):0.010503,((((NA18533.1:0.2055343776,(HG00684.1:0.2141285179,HGDP01231.0:0.1939691391):0.040770):0.027429,(HG00692.0:0.2033414197,HG00445.1:0.1906997580):0.058563):0.027522,(((((NA18975.0:0.1792653947,HG02153.0:0.1846615552):0.066853,HG00625.1:0.232842):0.015290,(NA18577.0:0.1814515549,HG00598.1:0.2024507885):0.049705):0.024803,((HG01841.1:0.1958778816,NA18618.1:0.2098552462):0.040147,NA18620.0:0.235619):0.023484):0.019334,((HG00611.0:0.2089410108,(HG00623.0:0.2035459237,HG00452.1:0.1925472660):0.039959):0.026136,(HG02088.1:0.1874322350,HG00671.1:0.2094616783):0.051188):0.026777):0.021054):0.016899,(((HG02082.1:0.270748,((HG02182.1:0.1715891404,HGDP01319.0:0.1896094475):0.052067,(NA18612.0:0.2083831951,NA19064.1:0.1969980078):0.056082):0.019596):0.018855,(((HG01029.0:0.1806541412,HGDP00719.1:0.2082373761):0.026109,HG02122.0:0.261008):0.033546,(HGDP01095.1:0.242112,(HG01801.1:0.1942548501,HG01798.0:0.1920347936):0.046817):0.028109):0.018754):0.017512,((((HG01859.1:0.1839900709,HG01869.0:0.1696945122):0.063414,HGDP01198.0:0.205539):0.016539,((HG02067.1:0.2023633159,HG02048.1:0.1996136343):0.049889,(HG02138.0:0.1751817054,HGDP01320.1:0.2059645251):0.040725):0.018067):0.027004,(HG01848.1:0.1794914483,HG00559.1:0.2045936849):0.067073):0.022108):0.017569):0.012368):0.006424):0.004472):0.005302):0.007378):0.004395,(((((((HG00542.0:0.1612997028,HG00614.1:0.1934905121):0.039005,(HG02050.0:0.2071210356,HG00632.0:0.1894327820):0.040136):0.025057,(NA18530.0:0.24005,HG02512.0:0.2151979642):0.019218):0.032782,(((NA19055.0:0.1842267336,HGDP01242.0:0.1686850574):0.050430,(NA18567.1:0.1823986968,NA18611.1:0.1710808601):0.048433):0.022664,((NA18976.1:0.210759,(NA18963.1:0.1721658922,NA18972.1:0.1869828133):0.050106):0.021858,(NA18984.0:0.1802060963,NA18972.0:0.1988366994):0.060510):0.027015):0.016870):0.014661,(((NA18963.0:0.1813598809,NA18964.1:0.2081406551):0.081462,(HG00610.0:0.1958614464,NA18991.1:0.1959331645):0.047185):0.027807,((NA18614.0:0.1707430914,HGDP01345.0:0.1855557709):0.037585,NA18631.1:0.2180917373):0.036529):0.024332):0.009893,((((((NA18638.0:0.1947673607,(HG00457.0:0.2010568032,NA19001.1:0.1726311979):0.043236):0.032936,((NA18641.1:0.1816510576,NA18645.1:0.1830210004):0.046265,NA18998.0:0.2241323182):0.027251):0.026154,((HG02156.1:0.2354478336,(HG01873.0:0.1904019701,NA18640.0:0.1904359820):0.039986):0.023897,(NA18619.1:0.2011910841,NA18610.1:0.1717205998):0.045376):0.028659):0.015982,((HG00560.1:0.2265034861,HG02180.1:0.247525):0.028182,(((HG01866.0:0.1805050101,NA18552.0:0.1908419300):0.041185,HG02116.1:0.1785863431):0.022216,HG01855.0:0.231436):0.034304):0.032033):0.016704,(((NA18564.1:0.1763159078,NA18950.1:0.2117856492):0.054021,(NA18537.0:0.1757960451,HG02190.0:0.1911409013):0.042151):0.018807,((NA18538.0:0.1797857139,HGDP01208.1:0.1747101280):0.032780,HGDP01346.1:0.2120764571):0.032793):0.034793):0.011190,((((HG00699.1:0.1841353172,HG00403.1:0.1875149547):0.041397,HG02069.1:0.2164450152):0.024399,HG01806.0:0.237658):0.031350,((HGDP01216.1:0.2266497119,(NA18629.1:0.1811912505,NA18534.1:0.1925688982):0.034622):0.028888,((HGDP01356.1:0.2003599526,HGDP01196.1:0.1760250743):0.040580,HGDP01356.0:0.2322152369):0.029018):0.018700):0.018072):0.010489):0.011565,((((((HG02383.0:0.1771228592,HG02371.0:0.1863501676):0.046012,(HG02406.0:0.2046871442,HG01031.1:0.1951064225):0.030633):0.023998,((HG00881.1:0.1586389805,HG00851.1:0.1390483798):0.062968,(HG00620.1:0.1780717564,HGDP01179.0:0.1790022414):0.062189):0.023677):0.026925,((NA18536.0:0.1824651008,HGDP01188.1:0.1728627904):0.059095,((HGDP01223.0:0.1931342040,HGDP01099.0:0.1700084375):0.049218,(HG01873.1:0.1884572658,HG02151.1:0.1826181137):0.056081):0.024568):0.028566):0.014673,((((NA18536.1:0.1846837193,HG00728.1:0.1855662915):0.053766,(HG02391.0:0.1592515655,HG02409.1:0.1654703199):0.081544):0.019365,(HG02028.0:0.1940051743,HGDP01183.0:0.2008158276):0.065091):0.027005,((HGDP01205.0:0.2026124991,HG02064.1:0.220573):0.033280,(HG02522.0:0.1882843315,HG00729.1:0.1789217141):0.075031):0.023343):0.014894):0.010531,(((((HG00463.1:0.1954301190,(HG00622.0:0.1861156022,HG01816.1:0.2001479641):0.042746):0.022911,(HG02035.0:0.1733810448,HG02355.0:0.1784593863):0.063994):0.021309,(NA18740.1:0.2055677116,HGDP00716.0:0.1802576535):0.055585):0.018205,HGDP01341.1:0.259):0.020970,(((HG02178.1:0.2288,NA18543.0:0.2025167749):0.029070,(NA18579.0:0.1929272048,HGDP01182.1:0.1985715759):0.048522):0.022190,((HG01797.1:0.1806692211,NA18623.0:0.1873272990):0.041548,HG02116.0:0.2157501071):0.034359):0.027272):0.033926):0.007803):0.010224):0.006627,((((((HGDP01103.1:0.255605,(NA18970.1:0.1799944441,HG02017.1:0.1793626620):0.062870):0.027597,(HG01868.0:0.23381,NA18561.0:0.2437522326):0.034902):0.027367,(((HGDP01337.1:0.1835058072,NA18646.1:0.1960405972):0.060935,HG00657.0:0.259031):0.024559,((HGDP01227.1:0.1926068281,HG00478.0:0.1620501571):0.053842,(HG00634.0:0.2060507374,NA18534.0:0.1691150110):0.041372):0.023789):0.021201):0.010985,((((((HG00689.1:0.1723653085,HG00698.0:0.1455721699):0.058256,HG00672.0:0.2015681859):0.035896,HG02180.0:0.2104832123):0.029384,(HG01860.1:0.1754575643,HG00445.0:0.1962794869):0.053716):0.029384,((HG02385.1:0.1854518215,HG00531.0:0.2004255457):0.040425,(HG02072.0:0.2117546509,(NA18985.0:0.1880996631,NA19005.1:0.1923271468):0.042723):0.032797):0.028663):0.019262,((((HG02079.0:0.1790025717,HG02137.1:0.1952884687):0.043474,(HG02360.1:0.1784591512,HG02131.1:0.2071870023):0.049892):0.026623,(HG02133.1:0.219336,HG02187.1:0.2376437892):0.037448):0.029681,(((NA18533.0:0.1983151001,NA19001.0:0.1831358607):0.044599,HG01861.0:0.2060227515):0.041314,((((HG00475.1:0.1457351262,HG00542.1:0.1349699626):0.056611,HG00500.1:0.1990208868):0.030621,(HG00651.0:0.2009136619,HG00428.0:0.1900227288):0.043703):0.028942,(HG02394.1:0.1966258836,HG00409.0:0.1864655625):0.066622):0.027423):0.018359):0.014601):0.007376):0.011481,((((HGDP00961.1:0.2263156906,HGDP01348.0:0.1907933778):0.054508,(HG02073.1:0.1848758831,HG00690.1:0.1891812703):0.042053):0.033404,((HG00693.1:0.1773560038,HG00428.1:0.2024415840):0.045029,HG00650.1:0.239954):0.020039):0.029317,(((NA18615.0:0.1925012625,HG00592.1:0.1850890211):0.055509,((HGDP01352.1:0.1821229935,NA18561.1:0.1769606485):0.042087,HGDP01342.1:0.2090639632):0.027914):0.030247,(HG01028.0:0.24233,(HG00464.1:0.2011335567,NA18596.1:0.1981560285):0.042805):0.029402):0.020489):0.016600):0.012247,(((((((NA18592.1:0.218658,(HGDP01095.0:0.1745208990,HG00537.0:0.2047345070):0.039522):0.020142,(NA18624.0:0.2105686964,HG02060.0:0.1866259907):0.060199):0.029320,((HG01848.0:0.256034,((HG00689.0:0.1476285343,HG00708.1:0.1777328772):0.060739,HGDP01222.1:0.2091150467):0.027598):0.027835,(HG02356.1:0.246323,(HG02165.1:0.2035658002,HG01871.0:0.1907143156):0.057761):0.021470):0.024563):0.014344,(((HGDP01212.0:0.1850150544,NA19000.1:0.1980037374):0.051179,(NA18566.0:0.2001316849,HGDP01220.1:0.1832411751):0.033615):0.020727,(HG00448.1:0.1922484770,HGDP01329.1:0.1944524181):0.065951):0.028275):0.012105,(((HG00619.0:0.1858587548,HG02139.0:0.1748400964):0.050794,(NA18608.1:0.1934222542,NA18548.0:0.1612928635):0.058986):0.021803,(HG00530.0:0.229207,HGDP00721.0:0.2238418449):0.041472):0.024747):0.012284,((((HG02356.0:0.1919845411,HG01850.0:0.1980247437):0.053641,(HG01845.0:0.1890523172,HG02152.0:0.2055988015):0.051537):0.024239,(HG00867.0:0.1713022428,NA18648.0:0.2106097136):0.063422):0.021565,((((HG02166.0:0.2015415644,(HG00978.1:0.1672209883,HG02185.0:0.1918813771):0.043918):0.037509,HG00699.0:0.219797):0.029103,((HG00449.0:0.1868660654,HG01804.1:0.1931109936):0.043167,NA18986.0:0.2131218232):0.032417):0.020169,((HG00446.1:0.2079104961,HG00844.0:0.1758965780):0.053074,(HG02181.1:0.1748067811,HG01802.0:0.1809931556):0.049847):0.035437):0.014914):0.023113):0.011282,(((((NA19089.0:0.2158812626,HG02060.1:0.1723851578):0.048358,NA18613.0:0.2158457811):0.035879,((HG01846.0:0.1972558324,NA18961.0:0.1749053386):0.056350,((HG02050.1:0.1934676468,NA18526.1:0.1985926748):0.055305,(HGDP01351.0:0.1915249413,HG00675.0:0.1744659593):0.059196):0.018274):0.019605):0.023269,((HG00610.1:0.1929472283,NA18757.1:0.2048130101):0.050253,((NA18535.1:0.1761692589,HG01029.1:0.2059220796):0.044303,NA18625.1:0.1908687658):0.029861):0.033636):0.013007,(((NA19005.0:0.2016852662,HGDP01230.0:0.1821723218):0.046305,(NA18642.1:0.1947257113,NA18745.0:0.1900440850):0.042961):0.018670,(HG01799.1:0.2010632816,(HGDP01326.1:0.1913040657,NA18627.1:0.1918023634):0.046849):0.040893):0.034821):0.016268):0.004572):0.012495):0.007180,(((((HGDP01236.1:0.1982226486,HG02398.1:0.1848953468):0.043222,NA18628.0:0.2294092035):0.052024,((((NA18547.1:0.2252875300,(HGDP01096.0:0.1900878776,NA18633.0:0.1882303273):0.036323):0.027003,(HGDP00720.1:0.2362606536,HG01849.0:0.1798482463):0.065218):0.023149,((HG02131.0:0.2116627290,HG02128.0:0.1962439730):0.054894,(HG00704.1:0.2089208913,HG02141.1:0.1925655789):0.050613):0.033609):0.018920,((NA18599.0:0.217399,(HG02019.0:0.1852207903,HGDP01346.0:0.2080077502):0.059014):0.030842,((HGDP01218.1:0.2162312897,(NA19064.0:0.1908117969,NA19065.1:0.2000816019):0.035931):0.025382,NA18559.0:0.228229):0.026286):0.021190):0.010845):0.008325,((NA18535.0:0.1973357015,NA19084.0:0.2019411995):0.070012,(((HGDP01212.1:0.2203605287,HGDP01221.1:0.229003):0.024277,(HG00653.1:0.2039250174,HGDP01101.1:0.1825571317):0.065984):0.024725,((NA18647.0:0.1922627845,HGDP01248.0:0.2223967812):0.037085,(HG00534.1:0.2002981131,NA18626.0:0.1766821570):0.048488):0.035729):0.019875):0.023471):0.014172,((((((HG01857.0:0.1765493438,NA18544.0:0.1979287288):0.034606,NA18528.1:0.2267673721):0.027612,(HGDP01355.0:0.2036250417,(NA18610.0:0.1875719845,HG00476.1:0.1888405469):0.049519):0.026600):0.022573,((HGDP01349.0:0.2131969628,HG02069.0:0.2029454820):0.08333,(HGDP01251.1:0.1888428281,NA18538.1:0.1906060027):0.053791):0.018020):0.017220,((NA18967.1:0.2207499312,((HGDP01225.0:0.2164662113,NA19058.0:0.1591284757):0.040890,(NA18987.1:0.1737648820,HG00978.0:0.1974705405):0.036019):0.025850):0.041247,(HGDP01226.0:0.249957,(HGDP01224.0:0.2281144068,(NA18947.0:0.2083081831,NA18553.0:0.1962772605):0.049119):0.035998):0.028360):0.010920):0.011879,(((NA19002.1:0.2013361212,NA18948.1:0.1912990376):0.058133,(HG01850.1:0.1997331513,HGDP01228.1:0.1984416189):0.042190):0.018874,NA18539.0:0.2499119863):0.045458):0.014363):0.012040):0.010262,((((((NA18993.0:0.1961022318,(NA19003.1:0.2023166974,NA19085.0:0.1807251961):0.056004):0.022152,(HG00607.0:0.1804123504,NA19011.0:0.1875637866):0.047195):0.038135,((((NA19089.1:0.1775944060,NA19085.1:0.2002111081):0.037832,NA18945.0:0.2032810352):0.033810,(NA19066.0:0.1777146180,NA19082.1:0.1882355740):0.051475):0.022166,(NA18995.1:0.218216,NA19063.0:0.2238757425):0.039528):0.022944):0.017591,HGDP00945.1:0.239759):0.020125,((((NA19087.1:0.233784,HGDP00950.0:0.2134527938):0.030275,((NA19009.1:0.1717768063,NA18979.0:0.2038044449):0.056271,NA19004.0:0.226204):0.028352):0.024257,(((NA18966.1:0.223774,HGDP01211.0:0.2314094323):0.030529,NA18989.1:0.233032):0.028218,(((NA18740.0:0.2013663700,NA19074.0:0.1738117249):0.044808,(NA19056.1:0.2051190911,HG00662.0:0.2097473276):0.045593):0.026011,(NA18952.1:0.2166494749,(HG00708.0:0.1999824199,NA18570.1:0.1717090553):0.038275):0.025709):0.019298):0.014533):0.014183,((((NA19079.0:0.2048848382,(NA18982.0:0.2025203693,NA19062.0:0.1760642661):0.042812):0.029997,(NA19090.0:0.1855811293,NA19078.0:0.1900670314):0.055482):0.012161,(NA18949.1:0.1903708003,HGDP00960.1:0.1901676140):0.046155):0.027166,((NA18967.0:0.1889650635,NA18960.1:0.2113765911):0.051198,((HG02410.0:0.236984,(HG00443.0:0.1916620023,HG02049.0:0.1992061797):0.046045):0.025884,(NA19057.1:0.1710718247,NA18985.1:0.1817307699):0.076574):0.016247):0.021961):0.016999):0.018060):0.010042,((((((HGDP01321.1:0.1030195817,HGDP01325.1:0.1458030380):0.081971,(HGDP01325.0:0.1275953888,HGDP01321.0:0.1144156293):0.081721):0.051217,(HG02392.0:0.2353618383,(HGDP01320.0:0.1938089101,HGDP01322.0:0.1701375523):0.040378):0.021757):0.024093,(HG00448.0:0.233729,HGDP00716.1:0.2420720914):0.041779):0.019606,(((((HGDP01191.1:0.1503059866,HGDP01191.0:0.1541427892):0.063745,HGDP01102.0:0.2212254642):0.031602,HGDP00721.1:0.2138101552):0.035661,(HGDP01351.1:0.2068171127,HG00956.0:0.1821707332):0.050709):0.020934,(HG01874.0:0.2125284156,HG02079.1:0.1807675609):0.064775):0.025609):0.014937,(((HG01864.1:0.2129121527,HG02394.0:0.1954096601):0.032704,(NA19066.1:0.1991374548,HG00611.1:0.1783777328):0.050553):0.036396,((HGDP01318.1:0.1763224304,(HGDP01322.1:0.1891940318,HGDP01318.0:0.1638732064):0.043369):0.044199,HGDP01317.1:0.2131795435):0.049382):0.028206):0.018489):0.008336):0.007330,((((((((NA18565.1:0.2079428149,HGDP01248.1:0.1848625056):0.044183,HG02133.0:0.2471693071):0.023437,(NA18647.1:0.1819574549,HGDP01245.1:0.1895911004):0.046311):0.011779,(NA19076.1:0.1918610471,HGDP01184.1:0.2045629824):0.060624):0.020362,((HGDP01204.1:0.2011651674,HGDP01209.0:0.1929397820):0.049751,HGDP01241.0:0.2180110031):0.037249):0.014665,((((HG02141.0:0.1955026287,NA18532.0:0.1704330637):0.047291,HGDP01352.0:0.2269560593):0.032532,((NA18980.1:0.1794581235,NA18964.0:0.1900283577):0.042340,HG00590.1:0.2090653120):0.029465):0.023352,(((HGDP01234.0:0.1832290638,HGDP01240.1:0.2001806373):0.050569,HGDP01237.1:0.2112990707):0.024880,(HGDP01204.0:0.1810993285,HGDP01249.0:0.1890780258):0.042591):0.026879):0.030639):0.011694,((((HGDP01220.0:0.1919653562,HGDP01213.0:0.1868176777):0.047428,(NA18555.1:0.1902178951,NA19080.1:0.1842344999):0.052594):0.021564,(((HGDP00950.1:0.2293803148,HGDP01205.1:0.1738920759):0.042839,(HGDP01229.1:0.1896665921,HG00421.0:0.1877363421):0.049700):0.042112,((NA18966.0:0.1928580691,NA18984.1:0.1881775005):0.063133,(NA19067.1:0.1820488133,NA19000.0:0.1934657869):0.051552):0.023167):0.026942):0.019099,(HGDP01194.0:0.259428,((NA18623.1:0.1890794472,HGDP01221.0:0.1672285845):0.038883,HG00650.0:0.2127433316):0.039252):0.026742):0.012091):0.011447,(((NA18982.1:0.2271972682,HGDP01206.1:0.212931):0.022512,(NA18961.1:0.1941397118,HGDP01104.1:0.1854611459):0.059777):0.028705,NA19060.0:0.280431):0.023291):0.009385):0.019219,(((((HGDP00947.1:0.1893955863,HGDP00956.1:0.1757210692):0.061911,(HGDP00965.1:0.2053342730,HGDP00955.1:0.1722239028):0.058372):0.016005,(HGDP00965.0:0.2006962867,HGDP01214.0:0.2101683851):0.048259):0.023249,((HGDP01240.0:0.1814657601,HGDP01234.1:0.1927268390):0.061226,(HGDP01235.1:0.1776807989,HGDP01242.1:0.1782864556):0.073645):0.026859):0.022352,(((HGDP01203.0:0.1185679971,HGDP01210.0:0.1047750790):0.107502,(HGDP01203.1:0.1177169959,HGDP01210.1:0.1238747862):0.103955):0.035205,((HGDP00945.0:0.1811887178,NA18747.1:0.2020267469):0.049531,HGDP01217.0:0.2109860895):0.031414):0.028230):0.023265):0.009624,(((((HGDP00952.0:0.2193089466,(HGDP00959.1:0.1779922881,HGDP00962.0:0.2032317588):0.039757):0.038634,((HGDP00948.0:0.2101009109,(HGDP00951.0:0.1669903991,HGDP00963.0:0.2012581528):0.041436):0.020287,((HGDP00966.1:0.1805520323,HGDP00954.0:0.1931913554):0.052798,(HGDP00947.0:0.1622009231,HGDP00956.0:0.1730598642):0.064098):0.018734):0.021387):0.022450,(((HGDP00959.0:0.1785872899,HGDP01211.1:0.2135795776):0.047150,(HGDP00960.0:0.1868397268,HGDP00955.0:0.2078643424):0.033219):0.029429,(HGDP00954.1:0.1989039116,HGDP00964.1:0.1968376363):0.064514):0.018689):0.016932,(HGDP01215.0:0.26381,(HGDP00964.0:0.2140889111,HGDP00966.0:0.2006530255):0.048487):0.044847):0.015947,((((HGDP01246.1:0.2270581293,HGDP00952.1:0.2232892948):0.039322,(HG00599.1:0.1952290046,HG00407.0:0.1775062846):0.051034):0.023052,((HGDP01347.0:0.1977753698,HG00404.1:0.1853226239):0.047859,HGDP00953.1:0.2339111565):0.044802):0.026351,((((HGDP01343.0:0.0889303301,HGDP01340.0:0.1374691142):0.097415,(HGDP01340.1:0.0953462337,HGDP01343.1:0.1385716235):0.082989):0.055268,(HGDP01229.0:0.2236790968,(HGDP00951.1:0.2066260929,NA19054.0:0.1797403813):0.038252):0.025354):0.028215,((HGDP01230.1:0.2283232704,HGDP01247.1:0.2222672554):0.044386,(HGDP01218.0:0.2144625429,HGDP01353.1:0.1964257488):0.045814):0.029632):0.019408):0.011963):0.016577):0.010743,((((HGDP00968.1:0.1666171084,HGDP00968.0:0.1597112648):0.065161,(HGDP00957.0:0.2171850415,HGDP00967.0:0.2030428357):0.033218):0.026900,(HGDP00948.1:0.1727643932,HGDP00957.1:0.2099647627):0.057922):0.031479,(((((HGDP00949.1:0.1941846532,HGDP00963.1:0.1891056718):0.047137,(HGDP00969.1:0.1989889161,HGDP00967.1:0.1839528687):0.038514):0.023429,(HGDP00946.0:0.2273843035,HGDP00962.1:0.2063160901):0.055163):0.019920,HGDP00946.1:0.2403079039):0.024093,(HGDP01243.1:0.267093,HGDP00949.0:0.2153213123):0.026511):0.020235):0.020038):0.025672,((HGDP01243.0:0.301672,(HGDP00953.0:0.2046357049,HGDP00958.1:0.1996022528):0.092924):0.014469,NA18642.0:0.299617):0.018487):0.147028,HGDP00969.0:0.329515):0.043616):0.028132,HGDP00961.0:0.460701):0.434488;
