# Supplementary figures and images for: Searching for Signatures of Cold Climate Adaptation in TRPM8 Gene in Populations of East Asian Ancestry
Source: Front Genet. 2019 Aug 23;10:759. doi: 10.3389/fgene.2019.00759 (PMC6716346; doi:10.3389/fgene.2019.00759)

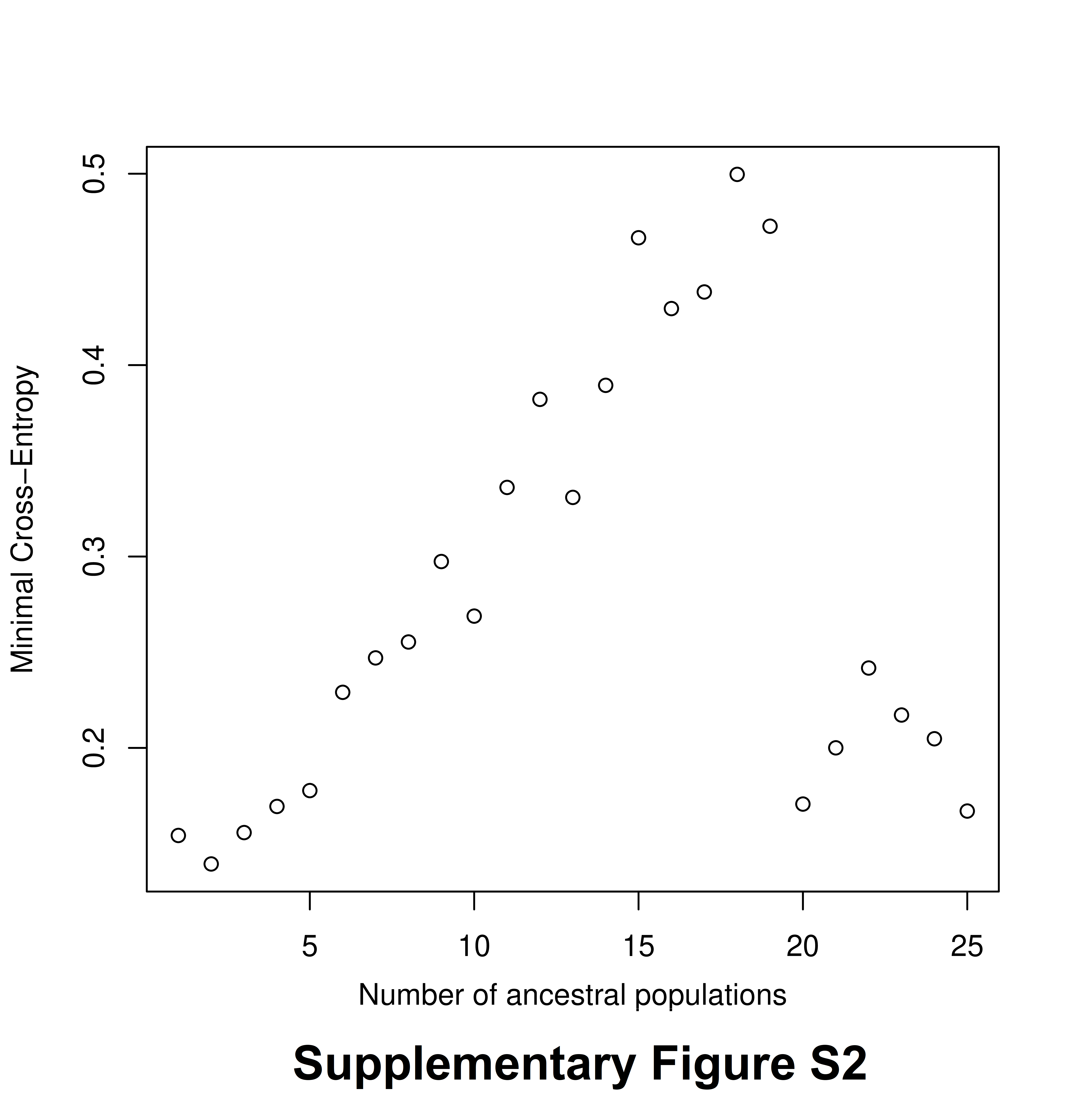

Supplement: Supplementary file 1 [file DataSheet_1.zip › SupplementaryMaterials/Supplementary Figure S2.tiff]
